# The consultant’s guide: evidence-based digital transformation framework for healthcare institutions in developing and resource-constrained economies

**DOI:** 10.1186/s12911-026-03713-9

**Published:** 2026-07-16

**Authors:** Uchechukwu Solomon Onyeabor, Okechukwu Onwuasoigwe, Wilfred Okwudili Okenwa, Omolade Ayoola Lasebikan, Thorsten Schaaf, Niels Pinkwart, Felix Balzer

**Affiliations:** 1 Federal University Oye-Ekiti, Oye-Ekiti, Nigeria; 2 University of Nigeria Teaching Hospital, Ituku-Ozalla, Nigeria; 3 ESUT Teaching Hospital Parklane, Enugu, Nigeria; 4 National Orthopaedic Hospital, Enugu, Nigeria; 5 Charité – Universitätsmedizin, Berlin, Germany; 6 Humboldt-Universität zu Berlin, Berlin, Germany

**Keywords:** Framework, Digital transformation, Developing countries, Healthcare, Digital adoption, Human-centered software engineering, Digital adoption, Design thinking, Agile

## Abstract

**Background:**

Digital transformation is increasingly reshaping healthcare delivery worldwide; however, the adoption of digital health systems in developing and resource-constrained economies remains constrained by infrastructural limitations, poor alignment with clinical workflows, inadequate user involvement, and resistance to organizational change. Many digital healthcare initiatives in these settings continue to experience implementation and sustainability challenges, partly due to the absence of context-specific and evidence-based transformation models. Consequently, this study aimed to develop a customized evidence-based digital transformation framework to support the sustainable adoption of digital healthcare systems within tertiary and university teaching hospitals in developing economies.

**Methods:**

This study emerged from a prolonged mixed-methods doctoral research project conducted across three tertiary healthcare institutions in southeastern Nigeria: the University of Nigeria Teaching Hospital (UNTH), ESUT Teaching Hospital (ESUTTH), and the National Orthopaedic Hospital Enugu (NOHE). The research involved close to 600 stakeholders, including doctors, nurses, medical records officers, patients, and clinical consultants. Data were collected through structured questionnaires, qualitative interviews, participatory co-design interactions, observational methods, usability testing, and iterative system evaluations. The study adopted an agile human-centered software engineering and participatory development approach to guide the design, implementation, and evaluation of the proposed TUMERIC digital healthcare support system and the resulting transformation framework.

**Results:**

The findings demonstrated generally positive institutional readiness toward digital transformation, with over 70% of participating healthcare professionals indicating support for future digital initiatives within their institutions. Usability evaluations of the implemented TUMERIC system also demonstrated high levels of user acceptance and perceived usability among clinicians and patients. Qualitative findings further highlighted the importance of aligning digital systems with existing clinical workflows, involving end users throughout the development lifecycle, and addressing infrastructural and organizational constraints during implementation. These findings informed the development of the proposed evidence-based digital transformation framework tailored to resource-constrained healthcare environments.

**Conclusion:**

The study demonstrates that agile human-centered and participatory design approaches can support the development and adoption of context-appropriate digital healthcare systems in developing economies. The proposed framework provides an empirically informed model that may assist healthcare institutions and policymakers in planning and implementing more sustainable and user-aligned digital transformation initiatives within tertiary healthcare settings.

**Supplementary Information:**

The online version contains supplementary material available at https://doi.org/10.1186/s12911-026-03713-9.

## Background

**Fig. 1:**
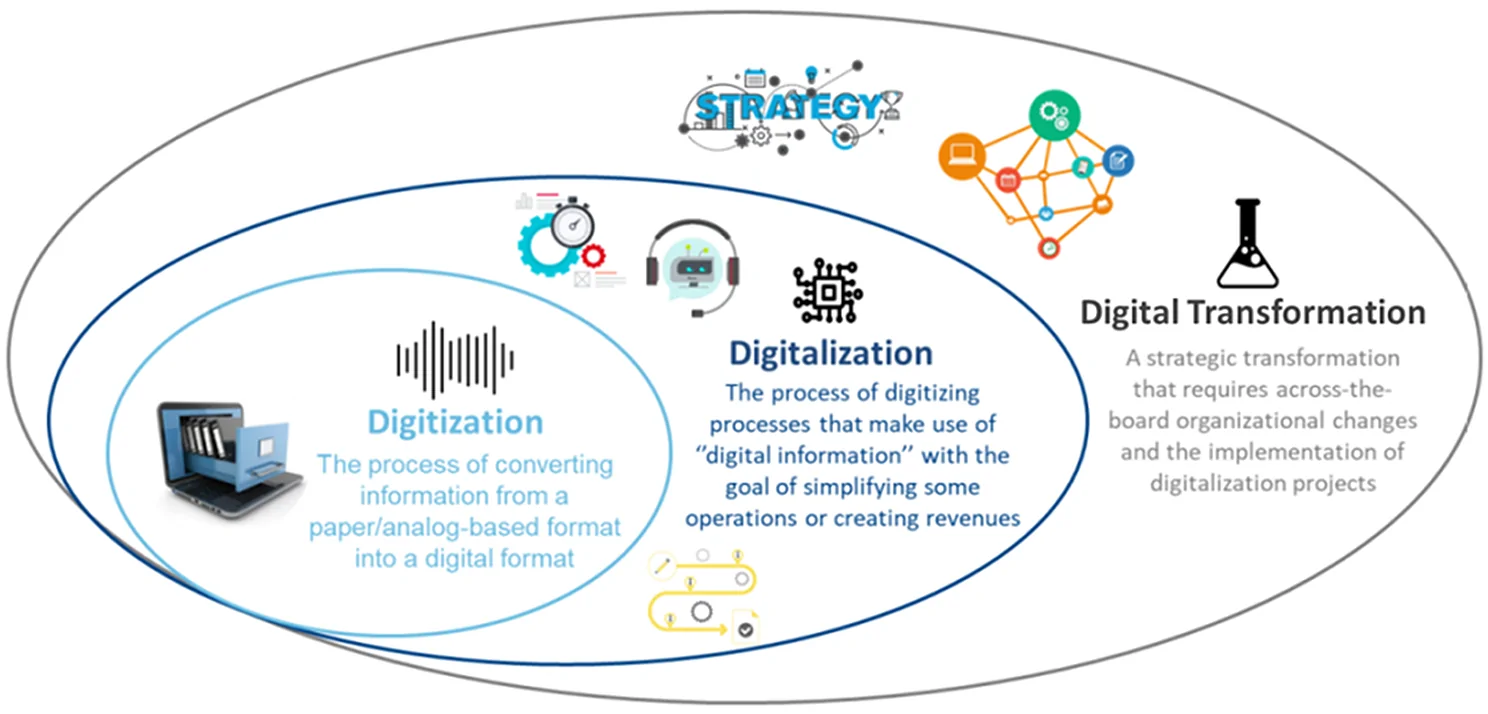
Digitization digitalization and digital transformation source: [4]

We are currently in a critical era, and several institutions are deploying strategies, procedures, and protocols, including a digital transformation campaign to sustain relevance, competitiveness, and efficiency [1]. The current wave of the COVID-19 global pandemic has further brought to the fore the urgency of the moment [2]. And as a result, the pressure on our healthcare system and institutions has suddenly become so massive that many of these healthcare providers barely cope with the sudden upsurge of demand on their facilities; some are already overwhelmed. So there is a need to transform! There is an urgent need to change the approach to healthcare delivery. There is a need for improved processes. There is a need to incorporate new measures, tools, and technologies that support service optimization and engender efficiency. By way of definition, digital transformation can be defined as a comprehensive process that requires organizations to rethink how they operate and interact with their customers. It involves the integration of digital technology into all areas of the business, from operations and marketing to customer service and product development [3]. These explanations are further conceptualized in Fig. 1 below.

The Fig. 1 in a healthcare context represents the progression from digitization converting paper-based medical records to electronic health records, to digitalization leveraging digital tools like AI, telemedicine, and remote monitoring to enhance healthcare processes, and finally to digital transformation, which entails a system-wide overhaul integrating digital technologies to improve patient care, streamline operations, and drive innovation in healthcare delivery. Digital transformation goes beyond just the implementation of new technology; it also requires a shift in culture and mindset. Organizations must be willing to challenge the status quo, experiment, and accept failure as part of the learning process [3]. Companies need to be open to new ideas and methods and embrace change as an opportunity to innovate and create value for their customers. As the digital landscape continues to evolve, organizations must remain agile and adaptive or risk being left behind [3].

### Digital transformation in healthcare

**Fig. 2:**
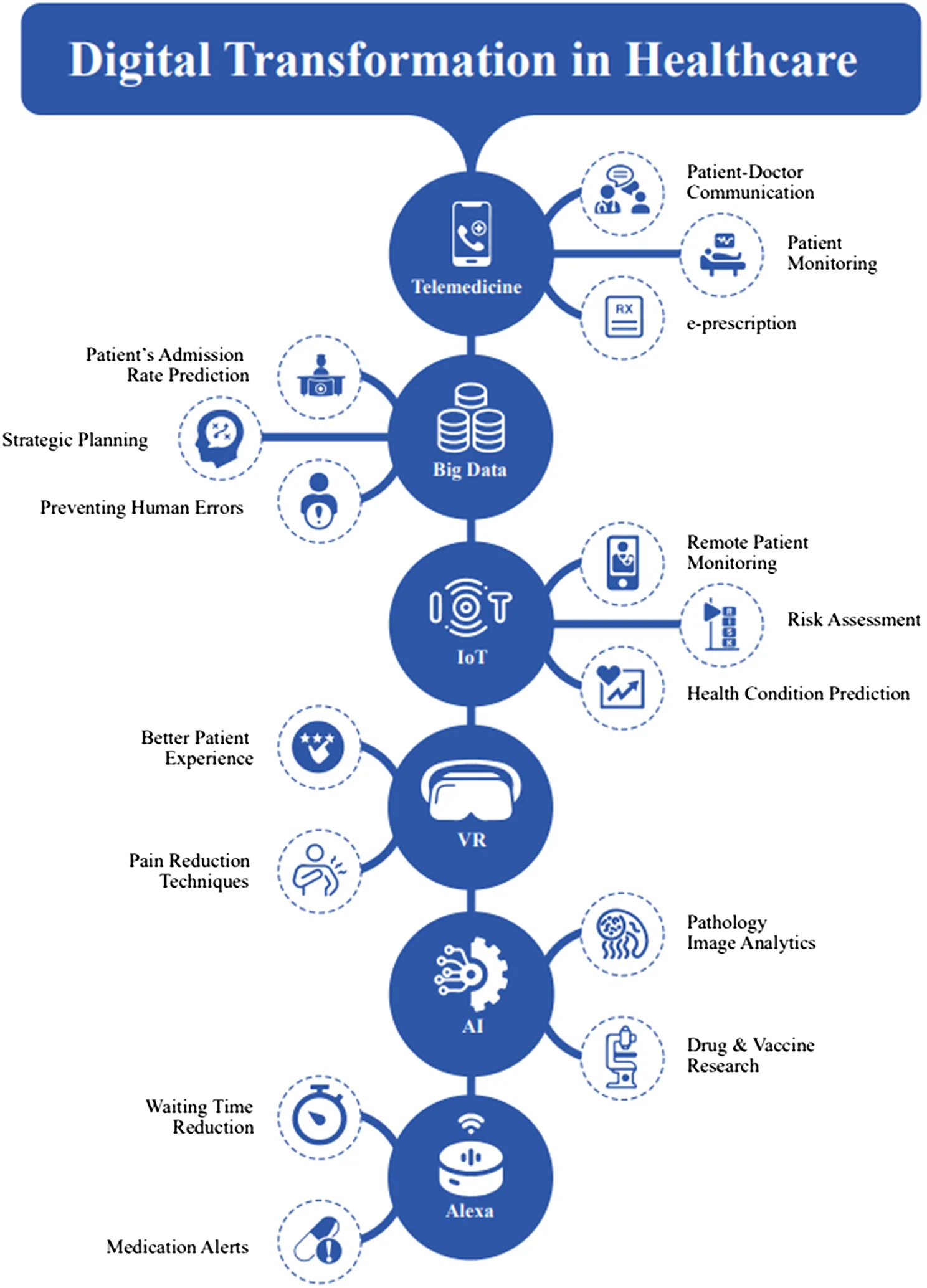
Digital transformation in healthcare source: [16]

Healthcare information technology is daily transforming the way and manner in which healthcare is administered to patients [5]. Digital transformation in the healthcare context can be considered the incorporation of digital and often emerging technologies in all aspects of healthcare service delivery. Digital technology has shown strong efficacy in improving the overall quality of health service delivery, ranging from the clinical workflow to patient health record management and the overall clinician and patient digital experience [6]. Therefore, digital transformation is an incredibly powerful tool for healthcare organizations, allowing them to revolutionize their approach to patient care, business strategy, and organizational processes [7]. By leveraging the latest advancements in information technology, healthcare organizations can harness the power of telehealth, predictive analytics, and artificial intelligence (AI) to gain a competitive edge [8]. Telehealth, for example, allows healthcare organizations to provide remote patient care and reduce the need for physical visits to the doctor’s office [9]. Predictive analytics, meanwhile, can be used to analyze historical patient data and predict future trends in healthcare, enabling organizations to proactively respond to the needs of patients before they even arise [10]. AI-powered technologies can be used to automate tedious and repetitive tasks, freeing up healthcare professionals to focus on delivering better care to their patients [11, 12]. With the right combination of technologies, healthcare organizations can unlock their full potential and revolutionize the way they deliver care [13]. As a result, countries around the world, like the United States of America, are investing huge resources in the form of billions of dollars and efforts towards the implementation of electronic health records systems [14]. In fact, healthcare policymakers look up to digital technology as a tool that could guarantee healthcare systems’ safety and ensure more accessible and affordable healthcare opportunities for the teeming population [15]. These emerging technologies and their potentials are further illustrated in Fig. 2 below. Figure 2 outlines key technologies driving digital transformation in healthcare, including telemedicine, big data, IoT, VR, AI, and Alexa. It highlights their applications, such as patient monitoring, risk assessment, pathology image analysis, drug research, pain reduction techniques, and medication alerts, all aimed at improving patient care, operational efficiency, and healthcare outcomes.

### Digital disruption in healthcare

**Fig. 3:**
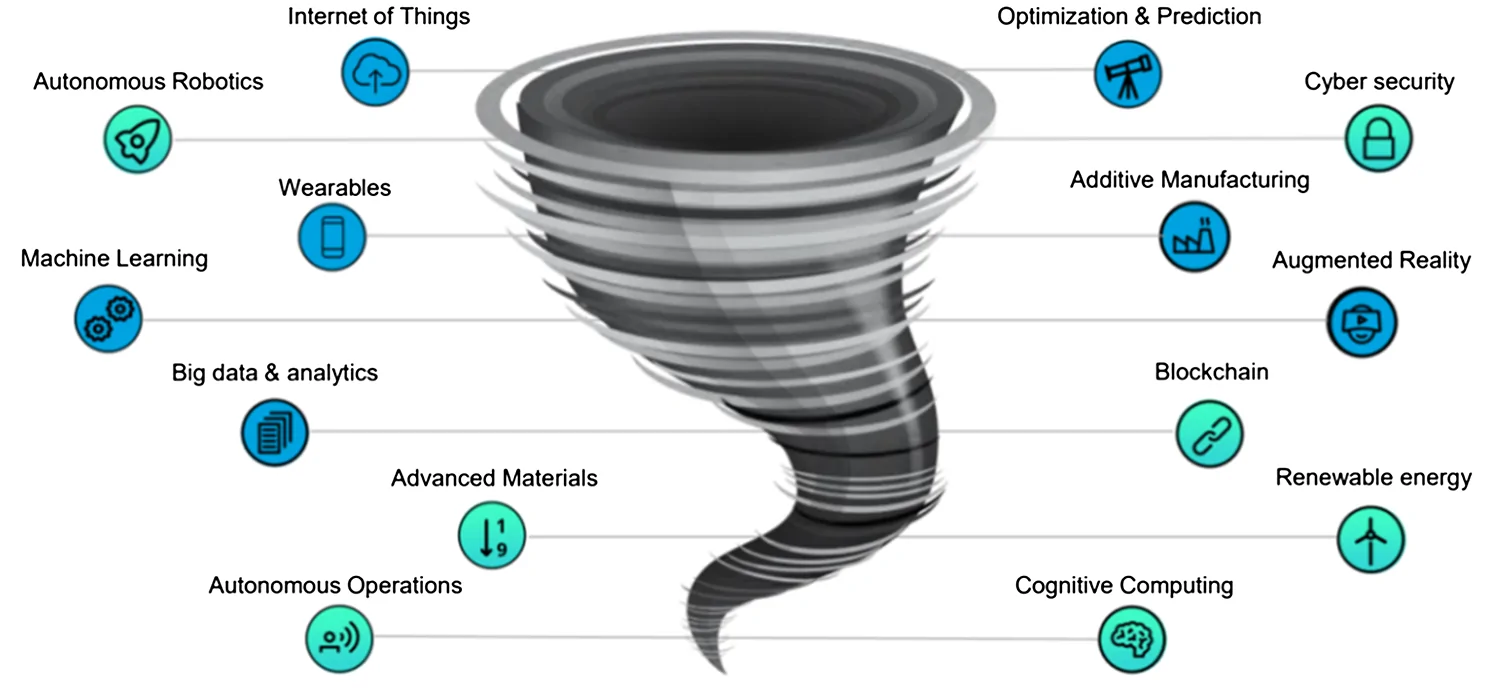
Emerging technologies disrupting healthcare ecosystem source: [20]

Digital disruption involves the introduction of new digital technologies to an industry, which brings about transformation, boosts effectiveness, and has the possibility to alter people’s lives significantly [17]. Regarding healthcare, we are observing a great deal of disruption in the form of AI being incorporated into care delivery [18] and have developed EHR systems that assist healthcare providers in enhancing care delivery by insisting that best practices are consistently communicated [19]. The healthcare sector is continually being disrupted by a variety of factors, key of which are changing consumer trends, emerging technologies, as shown in Fig. 3 below, and new players entering the industry [20]. Consumer preferences are constantly shifting and evolving, and as a result, companies must stay ahead of the curve in order to remain competitive. Furthermore, the rise of new technologies such as artificial intelligence, machine learning, and cloud computing has enabled healthcare providers to offer more personalized and efficient services [21]. Again, the arrival of new players in the industry, such as tech giants and venture-backed startups, is shaking up the traditional healthcare landscape and creating new opportunities for innovation [22]. All of these factors contribute to an ever-evolving healthcare sector that is ripe for disruption. Similar to Fig. 2 above, Fig. 3 below captures even more technologies currently disrupting the healthcare ecosystem. The image represents emerging technologies disrupting healthcare, including AI, machine learning, IoT, blockchain, AR, cybersecurity, and cognitive computing. These innovations drive advancements in patient monitoring, predictive analytics, robotic surgeries, precision medicine, and secure health data management, revolutionizing efficiency, accuracy, and patient care in the digital health era.

### Potentials and impact of emerging technologies in healthcare digital transformation

This segment of the literature review shall discuss the impacts of the various digital health intervention technologies, as shown in Fig. 2 above. Here we shall consider as follows: artificial intelligence, cloud computing, robotics, machine learning, blockchain technology, quantum computing, virtual reality, augmented reality, telemedicine, the internet of things, the internet of medical things, 5G, 3D printing technology, natural language processing, speech-to-text translation, autonomous vehicles, etc.

In summary, emerging technology like Cloud Computing (CC) is a powerful tool that enables healthcare providers to store and access vast amounts of data quickly and securely. It not only improves the efficiency of healthcare operations but also reduces costs [23]. The Internet of Things (IoT) is a network of connected devices and sensors that allow healthcare providers to collect and analyse data in real-time [24]. This can be used to improve patient care, monitor medical equipment, and automate administrative tasks. Artificial Intelligence (AI) enables healthcare providers to process data faster and more accurately than ever before, allowing for more informed decisions [25]. Again, Big Data (BD) is a powerful tool that helps healthcare providers identify trends and patterns in patient data, allowing them to make more informed decisions about treatment [26]. All of these technologies are essential for healthcare’s digital transformation, as they provide healthcare providers with the data and insights they need to stay competitive [27].

### Digital transformation frameworks and benefits

The Digital Transformation Framework is an essential tool deployed by modern businesses to achieve a successful and sustainable digital transformation campaign. The Digital Transformation Framework can be defined as an approach or methodology that enables organisations to leverage digital technologies and processes to enhance their products, services, and customer experiences [28]. A framework can be thought of as a roadmap for digital transformation, providing a clear path to success and enabling the implementation of technology to help achieve the desired goals [29]. It can be used to create an actionable strategy that outlines the steps needed to realise the desired outcome, such as which technologies should be used, how they should be used, and when they should be implemented [29]. Again, digital transformation frameworks provide businesses with a comprehensive roadmap of transformation strategies that are specifically tailored to their unique needs and goals [30]. By taking the guesswork out of the process, these frameworks offer businesses a clear path to success, outlining the steps they need to take in order to achieve their desired outcomes [30]. It helps to identify the areas for improvement and develop an action plan for implementing the necessary changes [30]. It also helps to identify the areas where the organisation needs to focus to become more efficient and competitive [30]. Furthermore, the digital transformation framework provides a clear roadmap for how to best utilise technology to increase productivity and efficiency [31]. Additionally, the digital transformation framework is a comprehensive guide to help organisations effectively develop their capabilities during the digital transformation process. It provides a holistic approach to digital transformation by outlining the different stages of the process and the essential elements required for successful implementation [32]. The framework helps organisations identify their digital objectives, assess their current capabilities, and identify areas for improvement. It also provides guidance on how to develop and maintain the necessary infrastructure, resources, and processes needed to drive digital transformation [32]. Additionally, the framework offers guidance on how to develop strategies and initiatives to ensure the successful integration of digital solutions into the organisation’s overall business operations [32]. Ultimately, the framework is designed to help organisations maximise the potential of digital transformation and ensure it is a successful endeavour.

Now the proposed evidence-based digital transformation framework was designed to support context-sensitive and sustainable digital adoption in healthcare settings, particularly within the context of Nigerian teaching hospitals. This framework aims to address the unique challenges faced by these institutions, such as resource constraints and the need for alignment with existing clinical workflows, by incorporating agile and human-centered design thinking principles. By providing a structured approach that involves all relevant stakeholders and emphasizes practical experience, the study contributes significantly to filling the knowledge gaps in the literature regarding digital health adoption, offering a tailored guide that reflects the realities of healthcare delivery in developing economies and with the potential to support improvements in healthcare delivery processes.

This study is positioned as an evidence-based framework development study grounded in mixed-methods empirical research and agile human-centered digital health system design. The proposed framework emerged from multiple interconnected research activities conducted across tertiary healthcare institutions in Nigeria, including organizational readiness assessments, clinician usability evaluations, qualitative investigations of implementation barriers, participatory requirements gathering, iterative system prototyping, and usability testing of the developed TUMERIC platform. The study therefore integrates empirical healthcare systems research with human-centered digital health intervention design to inform the development of a context-sensitive healthcare digital transformation framework for resource-constrained environments.

## Methods

### Research setting

This research was conducted across three tertiary healthcare institutions in southeastern Nigeria: the University of Nigeria Teaching Hospital (UNTH), Ituku-Ozalla; the ESUT Teaching Hospital (ESUTTH), Enugu; and the National Orthopaedic Hospital Enugu (NOHE). These institutions were selected because they represent major tertiary and referral healthcare centres within the region and provide diverse clinical environments for investigating healthcare digital transformation within resource-constrained settings.

The University of Nigeria Teaching Hospital (UNTH) serves as the primary teaching hospital for the University of Nigeria College of Medicine and has existed for over 60 years. Between 1991 and 2000, it was designated a World Health Organization (WHO) Centre for Innovation in Medical Education. Additionally, in 1986, the Federal Government of Nigeria recognized UNTH as a Centre of Excellence in Cardiothoracic Surgery. The hospital currently operates nine training programs and schools, encompassing 41 departments and three outposts, all within a 500-bed capacity. UNTH provides specialized and referral healthcare services across multiple disciplines, including family medicine, radiomedicine, orthopaedic and trauma surgery, gynaecology, cardiology, pharmacy, and ophthalmology, among others [33].

The ESUT Teaching Hospital (ESUTTH) originated in the 1930s and was formally upgraded to a state university teaching hospital in 2006. The hospital has an estimated capacity of 350–400 beds and serves as both a tertiary healthcare provider and a teaching institution for the ESUT College of Medicine. ESUTTH provides healthcare services across general medicine, obstetrics, paediatrics, surgery, physiotherapy, radiography, pharmacy, and laboratory diagnostics [34].

The National Orthopaedic Hospital Enugu (NOHE) was established by an Act of the National Assembly of Nigeria in 1979 and specializes in orthopaedic surgery, trauma surgery, burns and plastic surgery, physiotherapy, radiology, anaesthesia, and rehabilitation services. The institution also serves as a residency training centre for healthcare professionals and is one of Nigeria’s major specialized orthopaedic hospitals [35].

The broader research activities that informed the development of the proposed framework were conducted over several years as part of a doctoral research project investigating healthcare digital transformation and agile human-centered software engineering approaches within tertiary healthcare systems in developing economies.

### Research design

**Fig. 4:**
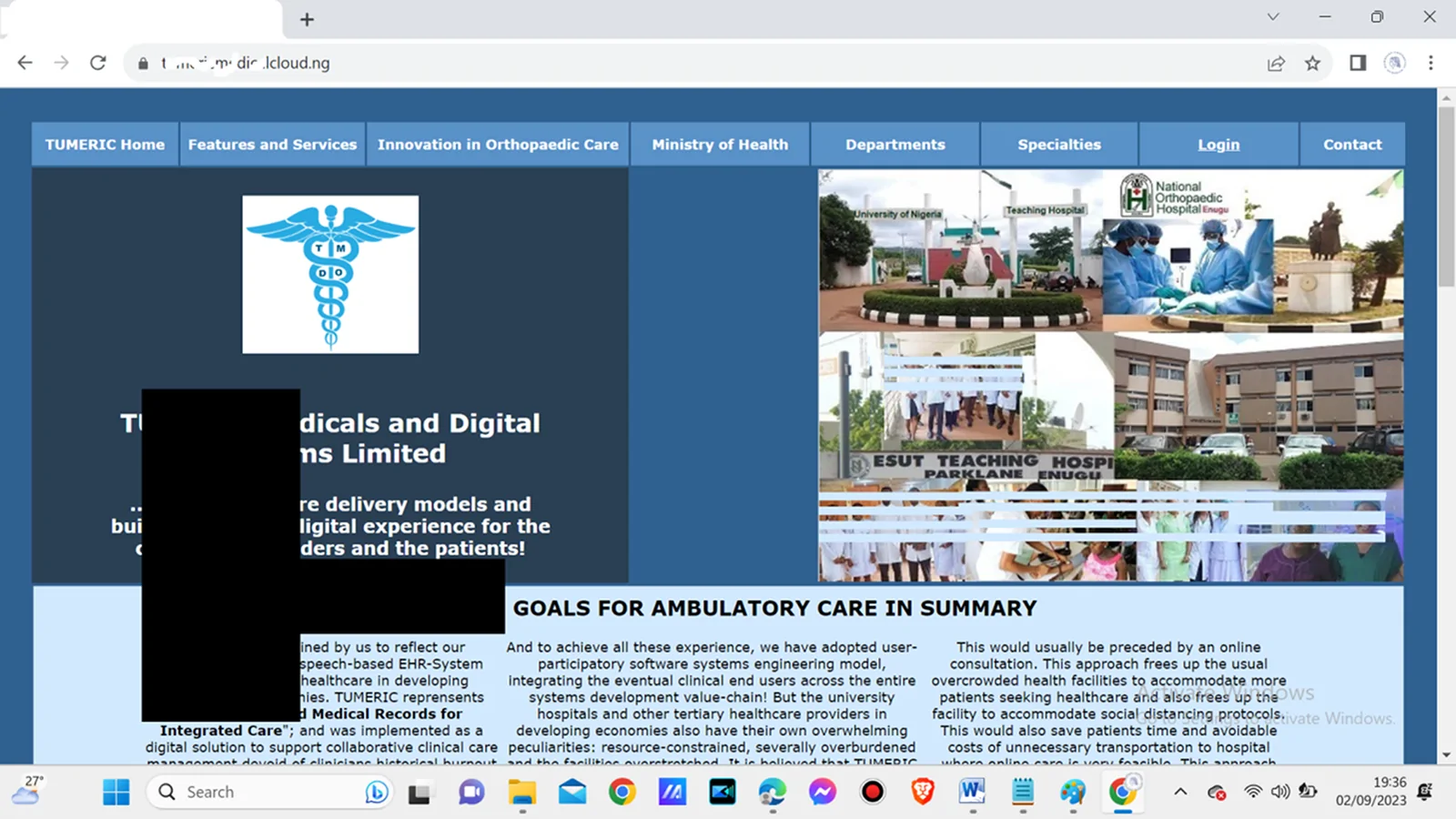
Homepage of the developed integrated cloud and voice-based clinical healthcare support systems

This study adopted a mixed-methods research design integrating quantitative, qualitative, participatory design, and system development methodologies. The broader research investigated the role of agile human-centered software engineering approaches in supporting healthcare digital transformation initiatives within tertiary healthcare institutions in Nigeria. Throughout the study, agile software development principles and human-centered design thinking paradigms guided the iterative design, implementation, and evaluation of the proposed cloud-based and voice-enabled healthcare support system. Meanwhile, an interface from one of the research outcomes is illustrated in Fig. 4 below.

The broader doctoral research project from which this framework emerged incorporated multiple interconnected empirical phases involving different participant groups, methodologies, and evaluation objectives. Consequently, the sample sizes reported across the study reflect distinct participant populations recruited for specific research activities, including organizational readiness assessments, clinician evaluations of existing digital systems, qualitative interviews regarding implementation barriers, participatory requirements gathering exercises, and usability evaluations of the developed TUMERIC platform. The present manuscript primarily focuses on the synthesis of findings most directly relevant to the development of the proposed evidence-based digital transformation framework rather than providing exhaustive reporting of all datasets generated across the broader research program.

The study incorporated quantitative surveys, qualitative interviews, direct non-participant workflow observations, focus group discussions, participatory requirements gathering activities, iterative prototype evaluations, and usability testing. The workflow observations involved direct observation of routine clinical and administrative processes, including patient registration, documentation practices, information flow, interactions with existing digital systems, and clinical workflow activities within the participating healthcare institutions. These methodological approaches were integrated to generate both empirical evidence and practical implementation insights capable of informing the development of the proposed evidence-based digital transformation framework.

**Fig. 5:**
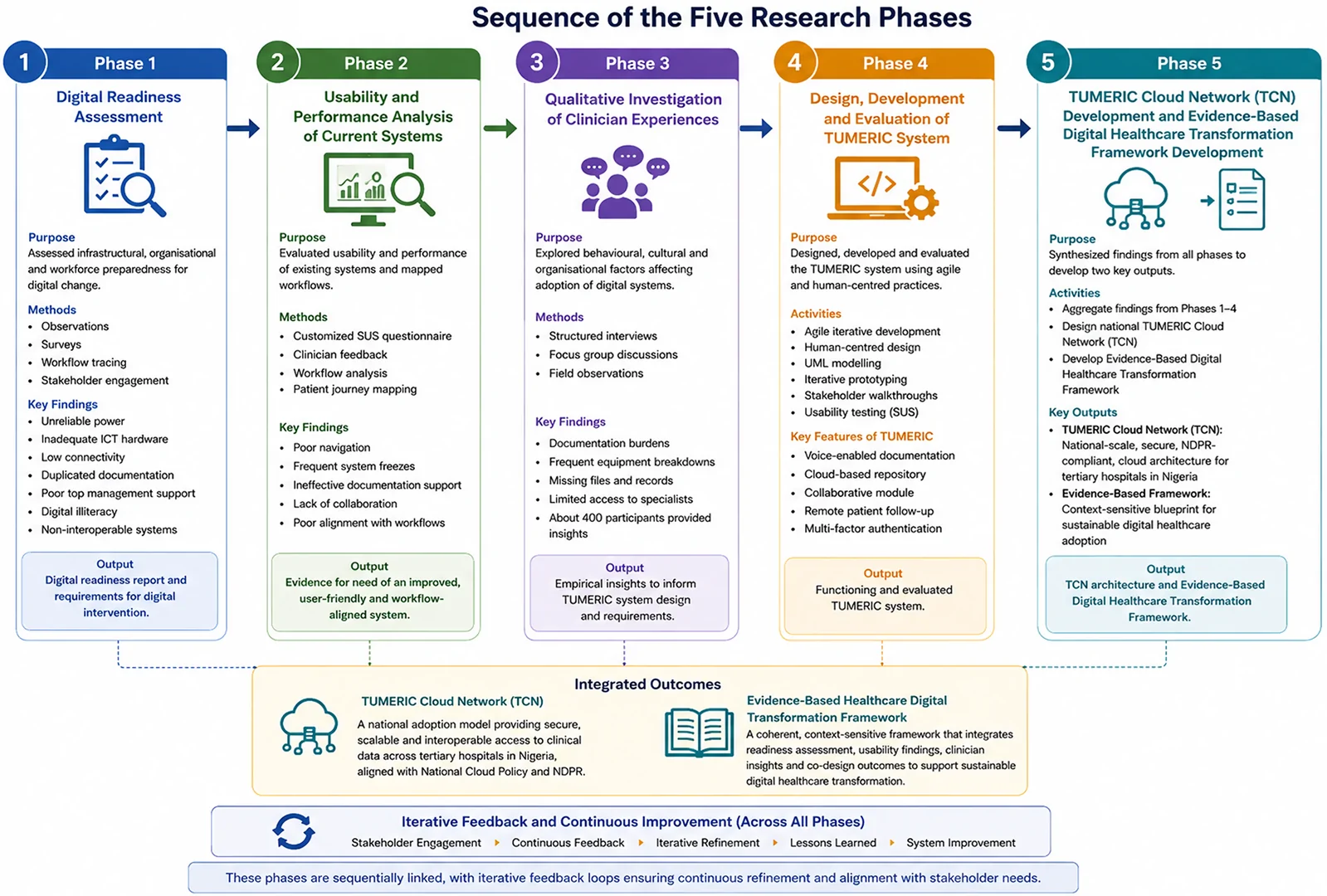
Sequence of empirical research phases undertaken in the development of the TUMERIC platform and the evidence-based healthcare digital transformation framework

Figure 5 summarizes the sequence of empirical research activities undertaken throughout the study. The research was conducted in five interconnected phases comprising organizational readiness assessment, evaluation of existing digital systems and workflows, qualitative investigation of stakeholder experiences and implementation barriers, iterative design and evaluation of the TUMERIC platform, and the synthesis of findings into the proposed evidence-based healthcare digital transformation framework. The outputs of each phase informed subsequent stages of the study through an iterative human-centered systems engineering process.

Accordingly, the methodological orientation of this study combined mixed-methods healthcare systems research with elements of design science and participatory digital health system development. The primary contribution of the study was the development of an empirically informed digital transformation framework grounded in iterative stakeholder engagement, organizational assessment, system design activities, and usability evaluation within real-world tertiary healthcare settings.

### Study population and sampling procedure

The study population comprised clinicians, healthcare administrators, patients, and medical records personnel working within the selected tertiary healthcare institutions. Participants included doctors, nurses, medical record officers, consultants, and patients who had direct interaction with existing or newly implemented digital healthcare systems within the participating hospitals.

Participants were selected using purposive and convenience sampling approaches depending on the specific phase of the research. Inclusion criteria included healthcare professionals actively involved in clinical workflows within the selected hospitals, clinicians with direct experience using existing electronic health record (EHR) or digital systems, stakeholders involved in healthcare documentation or patient management processes, and patients who interacted with the developed TUMERIC system during the usability evaluation phase. Individuals who had no interaction with digital healthcare systems or who declined participation were excluded from the study.

### Quantitative data collection

The first quantitative phase involved a survey assessing organizational readiness for digital transformation across the three tertiary healthcare institutions. The survey instrument was adapted from Paré’s organizational readiness model [36] and employed a 5-point Likert scale. The instrument consisted of 39 indicator variables designed to evaluate nine latent organizational dimensions, including vision clarity, change appropriateness, change efficacy, top management support, organizational flexibility, collective self-efficacy, organizational conflicts and politics, organizational history of change, and the presence of effective champions. A total of 580 healthcare professionals participated in this phase of the study.

The second quantitative phase examined clinicians’ experiences with existing digital healthcare systems. A structured 20-item questionnaire based on a 5-point Likert scale was administered to clinicians, including doctors, nurses, and medical record officers. The questionnaire focused on system usability, workflow compatibility, user satisfaction, documentation burden, interface design, and perceived technical limitations associated with the existing systems. A total of 131 clinicians participated in this evaluation.

### Questionnaire development and validation

The adapted usability evaluation questionnaire underwent expert review involving clinicians, healthcare researchers, and system development stakeholders to ensure contextual relevance and content validity. In addition, pilot testing was conducted prior to deployment to refine ambiguous items and improve clarity. The iterative adaptation process was undertaken to ensure that the evaluation instrument adequately reflected the operational realities and workflow requirements of the participating tertiary healthcare institutions.

Ethical approval for the study was obtained from the ethical review committees of the participating hospitals, ensuring compliance with institutional and research ethics requirements. Copies of the questionnaires and interview guides were uploaded as supplementary materials alongside the manuscript submission.

### Qualitative data collection

Qualitative data were collected through structured interviews, direct observations, and focus group discussions. The focus group discussions were conducted with purposively selected stakeholders drawn from the participating tertiary healthcare institutions. Participants included doctors, nurses, medical records officers, and ICT personnel who possessed direct experience with existing healthcare information systems and digital transformation activities. The focus group discussions followed a semi-structured format guided by predetermined questions relating to digital transformation readiness, implementation challenges, workflow processes, system usability, stakeholder expectations, and perceived barriers to adoption. The discussions also formed part of the requirements engineering process used to support the design, modelling, and subsequent development of the TUMERIC system. The discussions were documented through detailed field notes and subsequently incorporated into the qualitative thematic analysis alongside interview responses, workflow observations, and open-ended survey responses. Meanwhile, the structured interviews employed open-ended questions designed to explore clinicians’ perceptions regarding barriers affecting healthcare digital transformation implementation within tertiary healthcare institutions. Approximately 400 qualitative responses were obtained from participating clinicians across the three hospitals. The interviews explored issues relating to infrastructural challenges, organizational resistance, workflow disruptions, user experience concerns, training limitations, and implementation barriers associated with digital healthcare initiatives.

Direct observational methods were also employed to examine clinicians’ interactions with existing systems and workflows within routine clinical environments. The observations focused on system usability challenges, workflow inefficiencies, clinician frustrations, documentation practices, and user-system interactions. These observations provided contextual insights that informed the subsequent system design process.

Additionally, a focus group consisting of three senior consultant orthopaedic surgeons and departmental heads was established to support participatory requirements gathering for the proposed digital healthcare intervention system. The focus group sessions facilitated discussions relating to clinical workflow requirements, documentation challenges, communication needs, collaborative care processes, and essential system functionalities required within tertiary healthcare environments. The findings from these sessions informed the development of the TUMERIC cloud- and voice-enabled clinical support system.

### System development and iterative evaluation

The proposed digital healthcare support system was modelled using Unified Modeling Language (UML) techniques and developed iteratively following agile and human-centered software engineering principles. Repeated prototype evaluations and stakeholder feedback sessions were conducted throughout the development lifecycle to improve usability, workflow alignment, and overall user experience. The resulting system incorporated cloud-based and voice-enabled functionalities intended to support collaborative clinical workflows and healthcare documentation within resource-constrained healthcare settings.

### System usability evaluation

Finally, the completed system underwent another round of usability evaluation using an adapted usability evaluation questionnaire inspired by usability assessment concepts derived from the System Usability Scale (SUS) [37–39]. The instrument was modified to reflect context-specific clinical workflow requirements, perceived usability dimensions, and user interaction characteristics relevant to the developed cloud- and voice-enabled healthcare system. The questionnaire employed a 5-point Likert scale to assess perceived ease of use, workflow compatibility, learnability, interface usability, and overall user satisfaction among clinicians and patients who interacted with the developed system. Because the instrument was adapted for contextual applicability, the findings were interpreted descriptively rather than as standard SUS scores. A total of 20 patients participated in the TUMERIC usability assessment reported in this study. The clinician usability evaluation involved 75 healthcare professionals (30 doctors, 30 nurses, and 15 medical record officers) and has been reported elsewhere.

### Data analysis

Quantitative data obtained from the structured questionnaires and System Usability Scale evaluations were analyzed using descriptive statistical techniques, including frequency distributions, percentages, means, and comparative analyses across participant categories and hospital sites. Likert-scale responses were summarized using mean scores to evaluate organizational readiness, user experience, and perceived system usability.

Qualitative data obtained from interviews, observational activities, focus group discussions, and open-ended responses were analyzed using thematic analysis techniques. The qualitative responses were reviewed iteratively to identify recurring patterns, implementation concerns, workflow challenges, infrastructural barriers, usability issues, stakeholder expectations, and organizational factors influencing healthcare digital transformation initiatives.

The coding process involved the identification of preliminary categories and recurring concepts across the qualitative datasets, which were subsequently refined into broader thematic groupings aligned with the objectives of the study. The qualitative analysis focused primarily on identifying themes capable of informing the development of the proposed evidence-based digital transformation framework. To improve analytical consistency and contextual reliability, findings from interviews, observations, participatory engagements, and usability evaluations were triangulated during the synthesis process. Discrepancies in interpretation were resolved through repeated reviews of the qualitative responses and iterative discussions among the research team and participating clinical stakeholders.

### Framework development procedure

The proposed evidence-based healthcare digital transformation framework was developed through the synthesis of findings obtained from multiple interconnected empirical research activities conducted across the selected tertiary healthcare institutions. The framework development process followed an iterative and human-centered approach grounded in agile software engineering, participatory systems design, mixed-methods inquiry, and healthcare workflow analysis.

The organizational readiness assessment phase contributed to the framework components relating to institutional preparedness, leadership support, organizational change management, workforce readiness, and strategic digital transformation planning. Findings from the readiness surveys revealed variations in organizational readiness, vision clarity, change efficacy, top-management support, and collective self-efficacy across the participating institutions. These findings informed the inclusion of organizational readiness evaluation, leadership engagement, staff preparedness assessment, and digital transformation governance considerations within the proposed framework.

The clinician user experience and existing system evaluation phase informed framework dimensions relating to workflow alignment, system usability, interface design, interoperability considerations, and end-user involvement in digital transformation initiatives. The findings demonstrated recurring concerns regarding poor usability, workflow disruption, documentation burden, and inadequate alignment between existing digital systems and routine clinical processes. These observations reinforced the importance of incorporating workflow-centered design principles, usability evaluation, and continuous stakeholder participation into the framework structure.

The qualitative interview phase contributed significantly to the framework’s stakeholder engagement, implementation planning, and risk identification components. Clinicians and healthcare stakeholders identified infrastructural limitations, resistance to change, limited digital literacy, inadequate training, managerial challenges, and weak policy implementation as major barriers to digital transformation. These findings informed the inclusion of stakeholder engagement strategies, training and capacity development, infrastructural assessment, change management planning, and policy alignment mechanisms within the framework.

The focus group discussions and participatory requirements gathering activities informed the framework’s requirements elicitation and collaborative system development dimensions. Through iterative engagement with senior clinicians and departmental stakeholders, the research identified critical workflow requirements, documentation needs, communication processes, and collaborative care expectations necessary for designing context-appropriate digital healthcare systems. These findings reinforced the framework’s emphasis on participatory co-design and clinician involvement throughout digital transformation initiatives.

Furthermore, the iterative prototyping and usability evaluation phases informed the framework’s continuous evaluation and system refinement dimensions. The usability evaluation of the developed TUMERIC system demonstrated the importance of iterative testing, user feedback integration, and adaptive system improvement in enhancing user acceptance and perceived usability. Consequently, the framework incorporates continuous usability assessment, iterative refinement processes, and user-centered evaluation mechanisms as core implementation principles.

In addition to the empirical findings, the framework was further informed by existing literature, global digital health implementation principles, agile development methodologies, and human-centered software engineering theories. The integration of these empirical and theoretical inputs collectively informed the final structure of the proposed evidence-based healthcare digital transformation framework for tertiary healthcare institutions in developing and resource-constrained environments.

## Results

### Overview of the empirical research phases and datasets

The empirical findings presented in this study emerged from multiple interconnected phases of a broader doctoral research project investigating healthcare digital transformation within tertiary healthcare institutions in southeastern Nigeria. The study adopted a multi-phase mixed-methods and participatory human-centered systems engineering approach involving organizational readiness assessments, clinician evaluations of existing digital systems and workflows, qualitative investigations of implementation barriers and stakeholder experiences, participatory requirements elicitation activities, iterative prototype development, and usability evaluation of the developed TUMERIC platform. Different participant groups contributed to the various phases of the study based on the objectives and methodological requirements of each empirical component.

The organizational readiness assessment phase involved 580 healthcare professionals across the participating tertiary healthcare institutions. The clinician evaluation of existing digital systems and workflow processes involved 131 clinicians, including doctors, nurses, and medical record officers. The qualitative investigation phase generated approximately 400 open-ended responses relating to barriers affecting healthcare digital transformation implementation. Participatory requirements elicitation and co-design activities further involved senior clinical consultants, departmental stakeholders, and end-user representatives across multiple hospital units and departments. The final usability evaluation phase involved clinicians and patients who directly interacted with the developed TUMERIC cloud- and voice-enabled healthcare support system within routine clinical settings.

The findings presented in this manuscript primarily focus on the empirical results most directly relevant to the development of the proposed evidence-based healthcare digital transformation framework for tertiary healthcare institutions in developing and resource-constrained environments.

### Organizational readiness findings

The first empirical phase of the study assessed organizational readiness for healthcare digital transformation across the participating tertiary healthcare institutions. The assessment examined multiple organizational dimensions influencing institutional preparedness for digital transformation initiatives, including organizational vision clarity, leadership commitment, organizational flexibility, collective self-efficacy, change efficacy, infrastructural preparedness, workforce digital readiness, and organizational support for technological change.

The findings demonstrated moderate but variable levels of organizational readiness across the participating institutions. While many healthcare professionals expressed positive perceptions regarding the future adoption of digital healthcare initiatives, several institutional and infrastructural concerns were identified as potential barriers affecting implementation sustainability. Recurring concerns included inconsistent management support, inadequate digital infrastructure, funding limitations, insufficient digital literacy, unstable power supply, poor internet connectivity, excessive workload pressures, and resistance to organizational change.

Participants further emphasized the importance of strong leadership commitment, stakeholder engagement, institutional preparedness, staff motivation, and long-term organizational support structures in facilitating sustainable healthcare digital transformation initiatives. Collectively, these findings highlighted the importance of organizational readiness assessment, governance structures, infrastructural preparedness, workforce readiness, and strategic institutional alignment as foundational requirements for healthcare digital transformation within resource-constrained healthcare environments.

### Findings from existing digital systems and workflow evaluations

The second empirical phase of the study examined clinicians’ experiences with existing digital healthcare systems and workflow processes across the participating tertiary healthcare institutions. The findings revealed several technical, usability, infrastructural, and workflow-related limitations associated with previously implemented electronic healthcare systems.

Recurring concerns identified by clinicians included workflow disruption, excessive documentation burden, poor interface usability, inefficient system navigation, recurrent network failures, poor interoperability, inadequate technical support, and limited alignment between the implemented systems and existing clinical workflows. Several participants reported that previously implemented systems were “poorly designed” and “not consistent with our operational protocols,” while others described the systems as time-consuming within already high-volume clinical environments. Additional concerns included interrupted documentation processes caused by system failures, slow information retrieval processes, inadequate hardware infrastructure, and poor system responsiveness during routine clinical operations.

Direct observational activities similarly revealed practical challenges associated with healthcare documentation processes, delays in retrieving patient information, fragmented communication processes, and user frustrations during routine patient care activities. The findings further demonstrated that infrastructural instability, workflow incompatibility, and usability limitations significantly influenced clinicians’ perceptions regarding the effectiveness and sustainability of existing digital healthcare systems.

Collectively, these findings underscored the importance of workflow-sensitive system design, interoperability considerations, usability optimization, infrastructural reliability, low-bandwidth operational considerations, and human-centered implementation approaches during healthcare digital transformation initiatives within developing and resource-constrained healthcare environments.

### Qualitative findings from interviews and observational activities

The qualitative phase of the study explored stakeholders’ perceptions regarding barriers affecting healthcare digital transformation implementation within tertiary healthcare institutions. Approximately 400 open-ended responses, interview transcripts, observational notes, and focus group discussions were analyzed using a thematic qualitative analysis approach. Initial open coding was conducted independently by members of the research team to identify recurring concepts and implementation-related concerns emerging from the datasets. The preliminary codes were subsequently reviewed, refined, grouped into broader thematic categories, and reconciled through iterative discussions among the researchers to improve coding consistency and thematic alignment. The final themes were derived through consensus and mapped to the major components of the proposed evidence-based healthcare digital transformation framework.

The analysis revealed several recurring themes influencing the successful adoption and sustainability of digital healthcare initiatives within resource-constrained environments. Major themes included infrastructural limitations, workflow incompatibility, poor usability of existing systems, insufficient stakeholder involvement, inadequate training, digital literacy limitations, resistance to organizational change, and concerns regarding sustainability and technical support.

Participants frequently identified infrastructural limitations as major barriers affecting digital transformation initiatives. Recurring concerns included unstable electricity supply, poor internet connectivity, inadequate funding, and insufficient technical support capacity. These infrastructural and organizational concerns informed framework components relating to organizational readiness assessment, infrastructural preparedness, governance structures, and sustainability planning.

Another prominent theme concerned workflow disruption and poor alignment between existing digital systems and routine clinical practices. Several participants expressed dissatisfaction with the usability and operational inefficiencies of previously implemented electronic systems. One participant noted that “The current EHR was poorly designed, not consistent with our operational protocols was our major issue” [40]. Another respondent stated that “The existing system is time-consuming, and its use frustrates doctors’ daily routines” [40]. Similarly, another participant observed that “The EHR causes delays in our work. We end up focusing on the computer screen rather than the patient” [40]. Additional concerns were raised regarding documentation burden and inefficient system interaction, with one clinician explaining that “The current electronic system makes things very slow; typing the history is very cumbersome and slows things down” [40].

The findings also revealed concerns regarding implementation practices and insufficient stakeholder participation during previous digital initiatives. One participant stated that “EHR here is failing not because our staff is not ready, but because the EHR system introduced to us was inefficient.” [40]. These findings reinforced the importance of participatory stakeholder engagement, workflow-centered design, iterative usability evaluation, and human-centered implementation approaches during healthcare digital transformation initiatives.

Collectively, the qualitative findings highlighted the importance of context-sensitive implementation planning, participatory co-design, infrastructural preparedness, organizational support, and continuous user engagement during healthcare digital transformation initiatives within developing and resource-constrained healthcare environments. These empirical findings directly informed the development of the framework domains relating to organizational readiness and governance, participatory needs assessment and workflow analysis, human-centered system design and technical architecture, and deployment, sustainability, and capacity building.

#### Emergent qualitative themes informing the framework

The qualitative findings directly informed the conceptual structure and implementation dimensions of the proposed evidence-based healthcare digital transformation framework. Four major thematic areas emerged from the analysis: organizational and infrastructural readiness, workflow and usability challenges, stakeholder participation and organizational support, and iterative human-centered implementation practices.

Themes relating to infrastructural limitations, inadequate technical support, unstable internet connectivity, and funding concerns informed the framework dimensions associated with organizational readiness assessment, governance, infrastructural preparedness, and sustainability planning.

Similarly, recurring concerns regarding workflow disruption, inefficient documentation processes, usability limitations, and poor alignment between existing systems and routine clinical practices informed the inclusion of workflow-centered and human-centered design principles within the framework. For example, one participant noted that “The current EHR was poorly designed, not consistent with our operational protocols was our major issue” [40]. Another participant stated that “The EHR causes delays in our work. We end up focusing on the computer screen rather than the patient” [40].

Themes associated with inadequate stakeholder engagement, insufficient training opportunities, and weak organizational support structures informed framework components relating to participatory implementation, training and capacity development, and organizational change management. Additionally, findings relating to iterative usability evaluation and participatory co-design informed the framework’s emphasis on continuous system refinement and agile human-centered implementation processes.

Collectively, these qualitative themes provided empirical support for the framework domains relating to organizational readiness and governance, participatory needs assessment and workflow analysis, human-centered system design and technical architecture, and deployment, sustainability, and capacity building.

### TUMERIC system development and usability evaluation findings

The findings obtained from the organizational readiness assessments, workflow evaluations, qualitative investigations, and participatory requirements elicitation activities informed the iterative development of the proposed TUMERIC cloud- and voice-enabled collaborative healthcare support system. The system was developed using agile human-centered software engineering principles involving iterative co-design, UML-based system modelling, repeated stakeholder feedback cycles, and continuous prototype refinement throughout the system development lifecycle.

The completed system subsequently underwent usability evaluation using an adapted usability evaluation questionnaire informed by System Usability Scale (SUS) principles [37–39]. The usability evaluation involved clinicians and patients who directly interacted with the developed system within routine clinical environments.

The usability findings demonstrated generally positive perceptions regarding system learnability, ease of use, workflow compatibility, interface usability, accessibility, and overall user experience associated with the implemented healthcare support platform. Participants further reported positive perceptions regarding the collaborative functionalities, usability simplicity, and practical applicability of the proposed cloud- and voice-enabled healthcare support system within routine clinical workflows.

The iterative usability evaluation activities additionally demonstrated the importance of participatory co-design, continuous stakeholder engagement, iterative refinement processes, and workflow-sensitive system development approaches during healthcare digital transformation initiatives. However, the findings should be interpreted cautiously, as the present study did not employ a comparative experimental design capable of establishing causal relationships between the adopted agile human-centered development methodology and the observed usability outcomes.

**Fig. 6:**
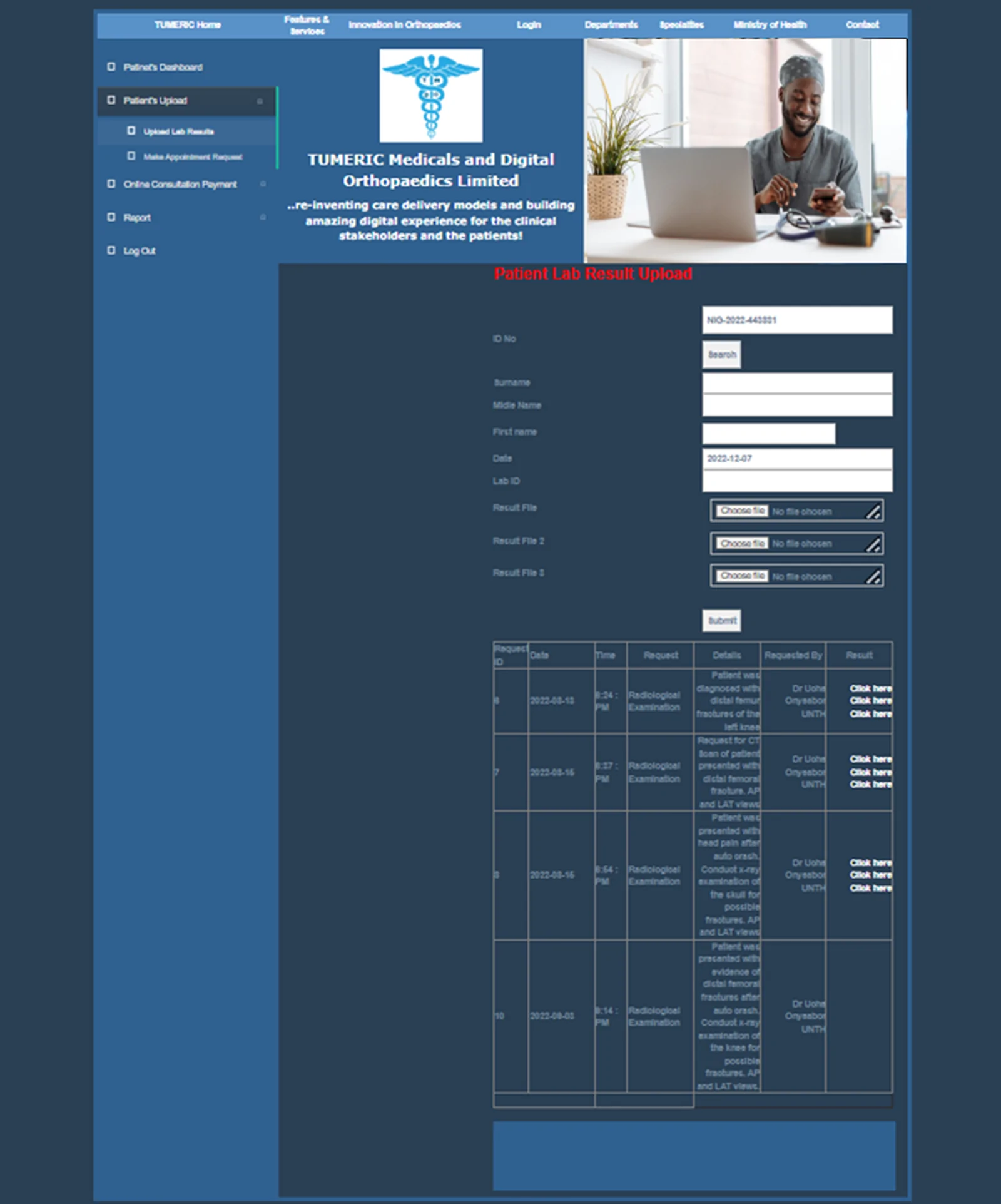
Patient dashboard on the implemented digital healthcare support system showing the lab/radiological reports and images upload

**Fig. 7:**
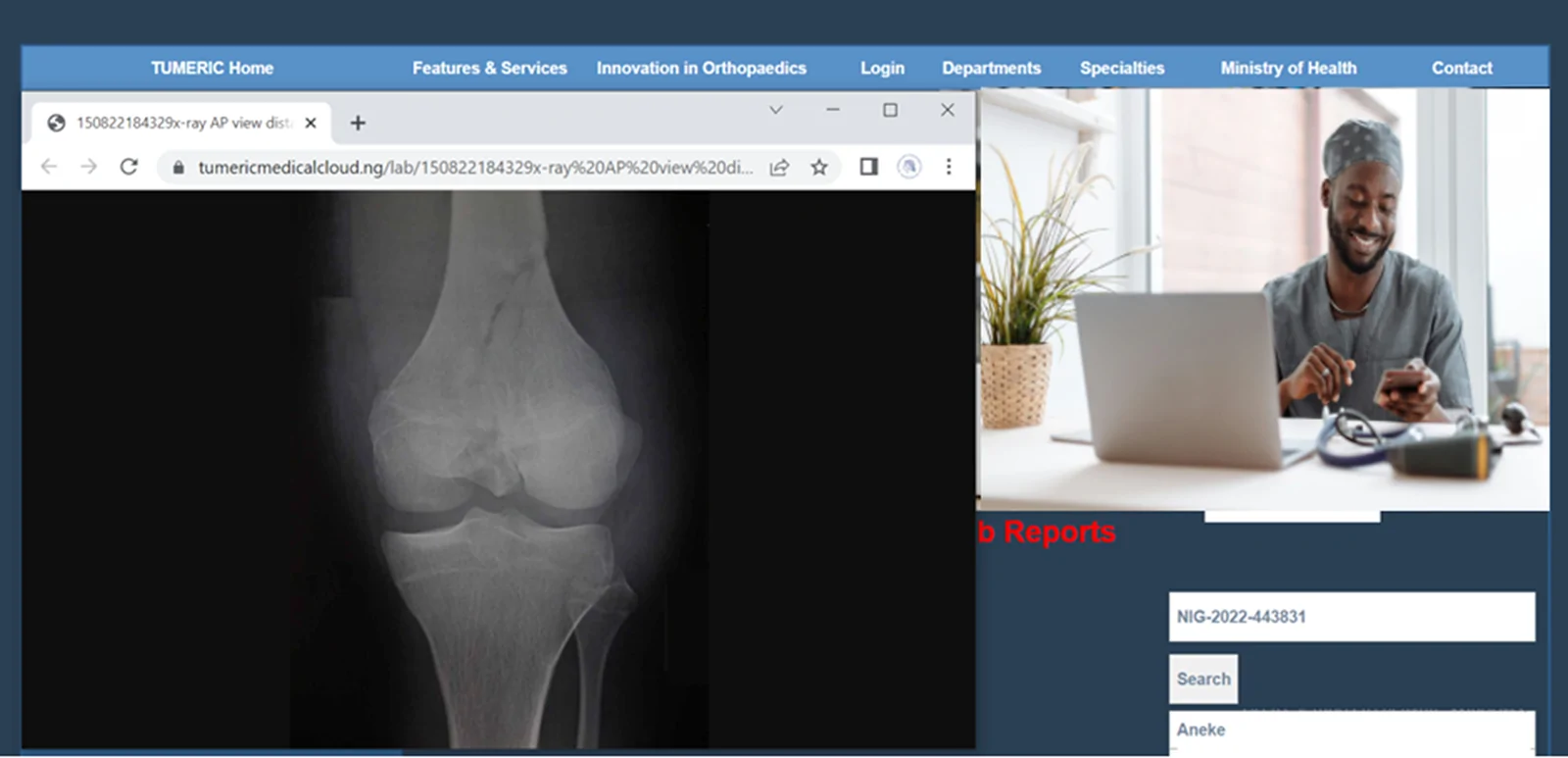
Doctor’s anteroposterior view of the knee on implemented digital healthcare system

Meanwhile Fig. 6 presents the interface of the patient dashboard on the implemented digital healthcare support system showing the lab/radiological reports and images upload and Fig. 7 the screenshot of the doctor’s anteroposterior view of the knee on the same system system.

**Fig. 8:**
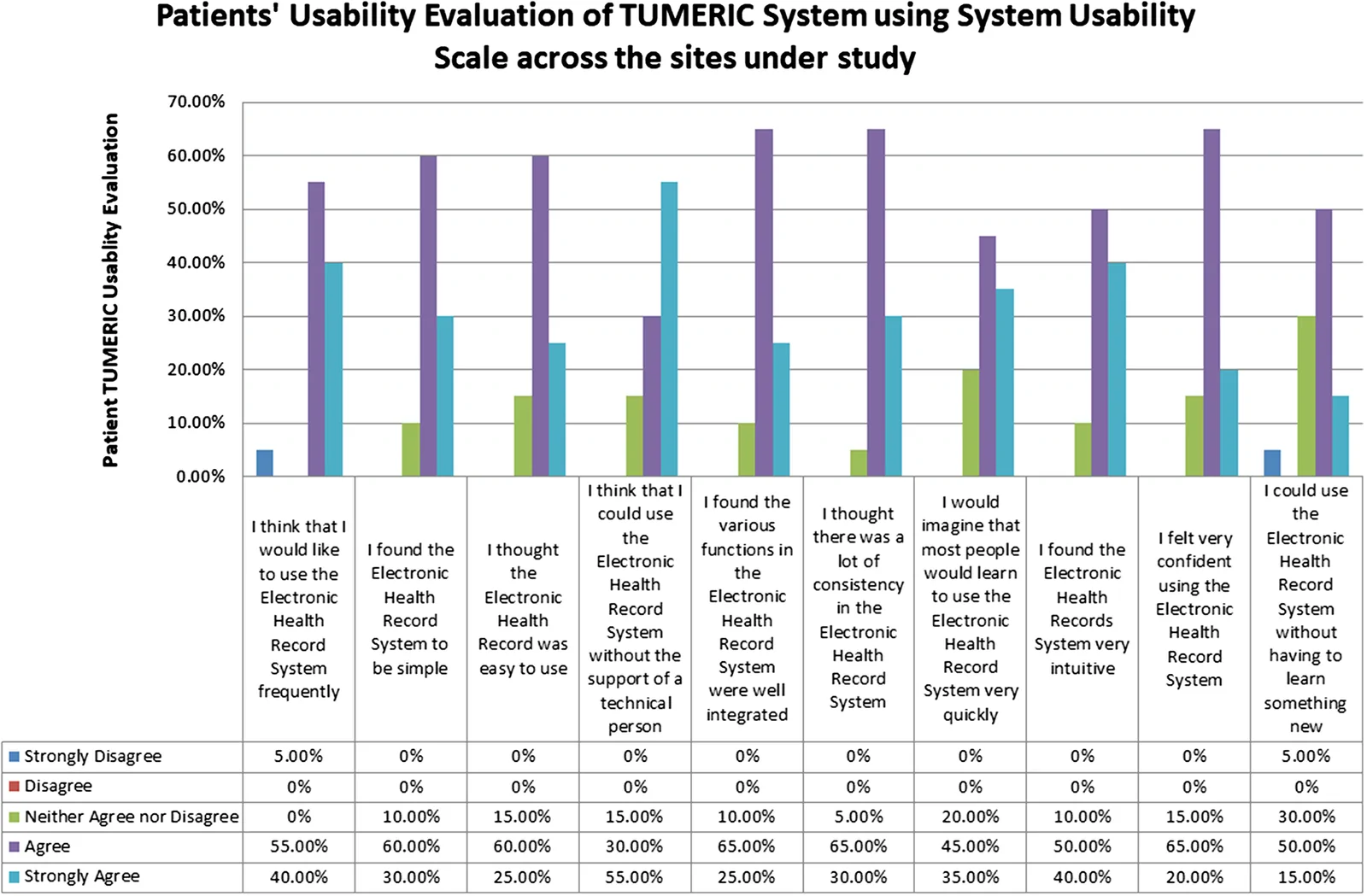
Patients’ usability evaluation of implemented system based on an adapted usability evaluation questionnaire informed by System Usability Scale (SUS) principles

Meanwhile Fig. 8 presents the result of the Patients’ usability evaluation of TUMERIC system based on an adapted usability evaluation questionnaire informed by System Usability Scale (SUS) principles.

### Framework synthesis and development

The proposed evidence-based healthcare digital transformation framework emerged from the cross-phase synthesis and integration of findings obtained throughout the multiple empirical phases of the broader doctoral research project. The framework components were informed by organizational readiness assessments, clinician workflow evaluations, qualitative stakeholder experiences, participatory requirements elicitation activities, iterative prototype development processes, usability evaluations, and practical implementation experiences generated during the development and evaluation of the TUMERIC platform.

The organizational readiness findings informed framework dimensions relating to institutional preparedness assessment, leadership engagement, governance structures, organizational change management, workforce readiness, infrastructural preparedness, and strategic digital transformation planning. Findings from the clinician workflow evaluations informed the inclusion of workflow alignment mechanisms, human-centered design principles, interoperability considerations, usability optimization processes, and continuous system evaluation strategies within the framework structure.

Similarly, the qualitative findings relating to infrastructural instability, stakeholder concerns, digital literacy limitations, resistance to organizational change, inadequate technical support structures, and implementation sustainability challenges informed framework components associated with participatory stakeholder engagement, implementation planning, training strategies, infrastructural assessment, organizational adaptation, and long-term sustainability planning.

Furthermore, the iterative prototyping and usability evaluation activities reinforced the importance of participatory co-design, iterative system refinement, continuous usability evaluation, agile human-centered software engineering approaches, and workflow-sensitive implementation methodologies during healthcare digital transformation initiatives within resource-constrained healthcare environments.

**Fig. 9:**
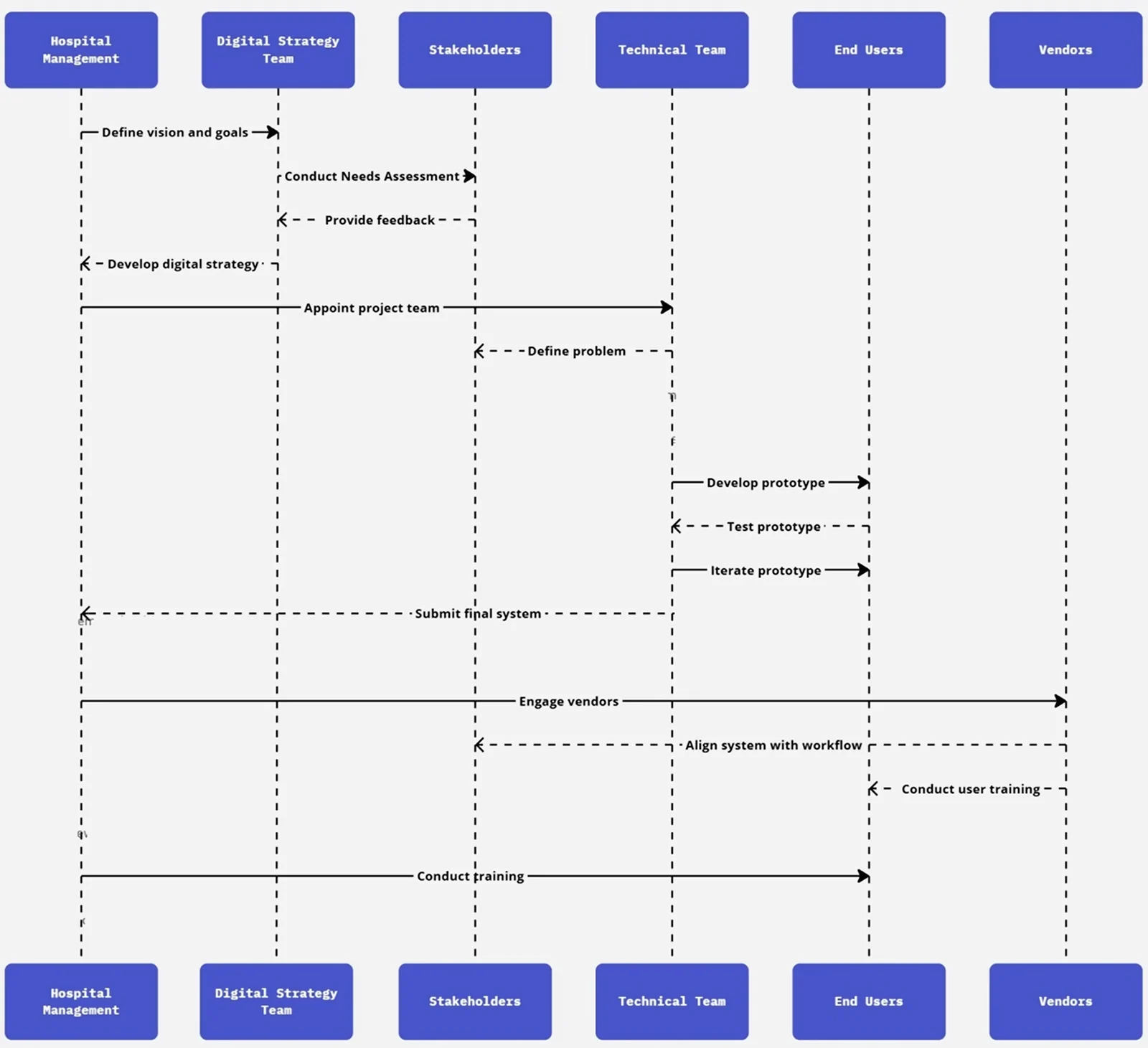
Sequence diagram of the empirically derived healthcare digital transformation implementation framework

**Fig. 10:**
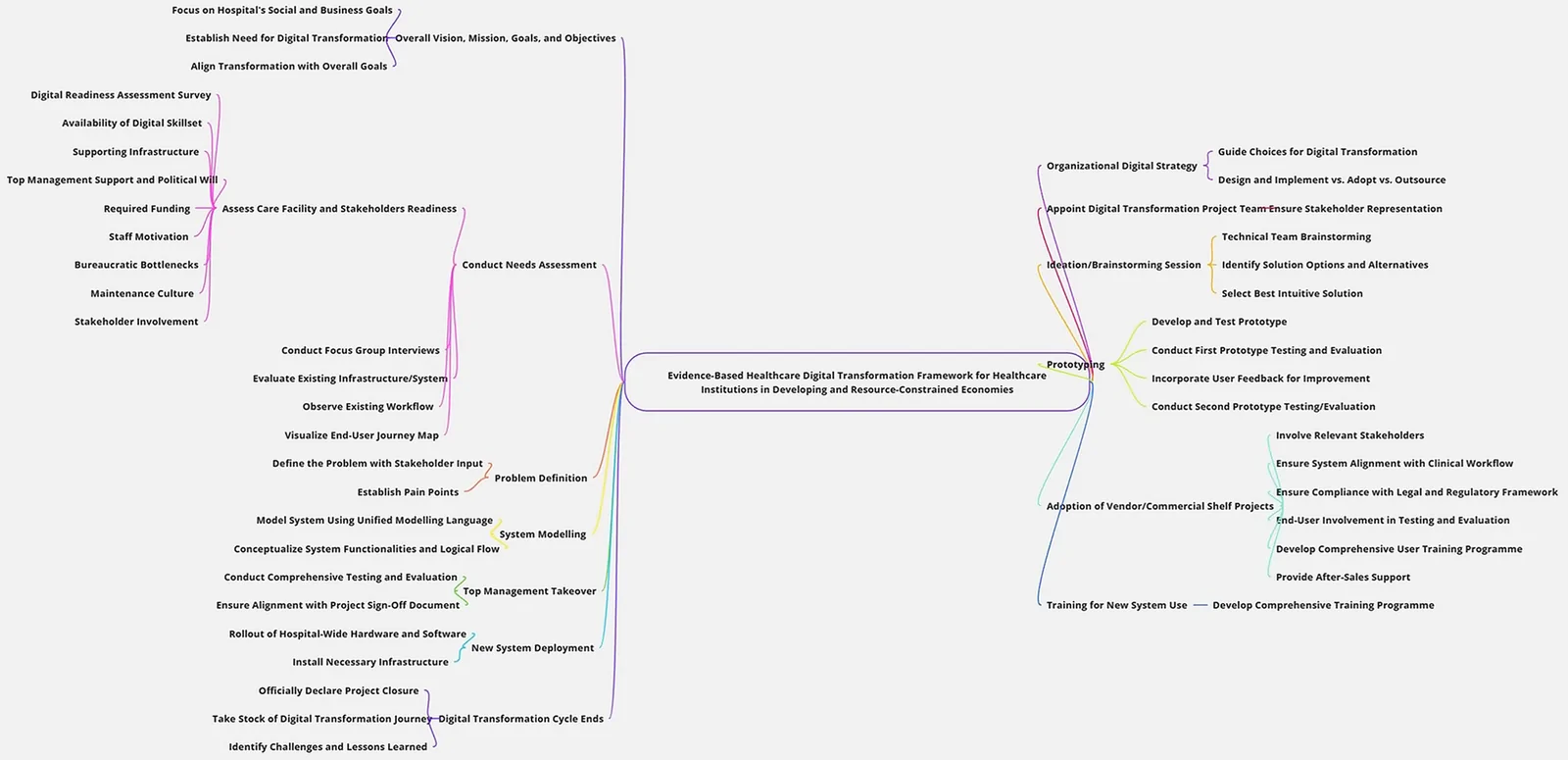
Mind Map of the empirically derived healthcare digital transformation implementation framework for healthcare institutions in developing and resource-constrained economies

Table 1 presents the high-level evidence-based healthcare digital transformation framework synthesized from the empirical findings, while Table 2 presents the field-based operational representation of the framework derived from the practical implementation experiences and participatory development activities conducted throughout the study. Figures 8, 9 and 10 further illustrate the conceptual structure, operational relationships, and implementation flow of the proposed framework.

**Table 1:** High-level evidence-based healthcare digital transformation framework for healthcare institutions in developing and resource-constrained economies

Framework Domain	Core Components	Empirical Basis from Study
1. Organizational Readiness and Governance	Alignment of digital transformation with institutional vision, strategic digital planning, organizational readiness assessment, leadership commitment, stakeholder representation, funding considerations, change management readiness, infrastructural preparedness, digital literacy assessment, policy alignment, regulatory and legal compliance planning	Informed by institutional readiness assessment findings, leadership variability, infrastructural constraints, and clinician perceptions identified during Phases 1 and 3
2. Participatory Needs Assessment and Workflow Analysis	Stakeholder engagement, clinical workflow observation, end-user journey mapping, identification of pain points, evaluation of existing digital systems, clinician interviews, focus group discussions, participatory requirements elicitation, assessment of interoperability requirements, evaluation of legacy systems and data migration needs	Derived from workflow observations, clinician experience assessments, qualitative interviews, and focus group findings obtained during Phases 2 and 3
3. Human-Centered System Design and Technical Architecture	Agile and HCSE-based iterative development, ideation and brainstorming sessions, UML-based system modelling, prototype development, iterative usability testing, interoperability considerations, standards-based APIs and integration protocols (e.g., HL7/FHIR), integration with existing EHR systems, cloud-based deployment assumptions, authentication and authorization mechanisms, role-based access control, privacy and cybersecurity considerations, auditability, low-bandwidth optimization, scalability, NDPR-compliant architecture, maintenance planning, backup and disaster recovery considerations, data harmonization and migration planning	Informed by participatory co-design activities, UML modelling, iterative prototyping, usability evaluations, and technical architecture development undertaken during Phase 4
4. Deployment, Capacity Building, and Sustainability	Management evaluation and approval, staged deployment, user training programs, technical support structures, post-deployment usability evaluation, continuous system refinement, long-term maintenance, sustainability planning, institutional adaptation, implementation monitoring, operational cost planning, cloud hosting and infrastructure sustainability, maintenance and technical support budgeting, sustainability and funding strategy	Derived from implementation experiences, stakeholder feedback, usability evaluations, and iterative refinement processes across Phases 4 and 5

**Table 2:** Empirically-derived healthcare digital transformation implementation framework for tertiary healthcare institutions in resource-constrained settings

**1.** Keep the overall vision, mission, the goals and objectives of the hospital in focus. Your digital transformation drive must be in the context of your hospital overall social and business goals. *Establish why your hospital really need to embark on this digital transformation journey and how this exercise would help with your overall social and business goals*	**5.** Problem Definition: With outcome of earlier research instruments and working with stakeholders define the problem; establish the pain points.	**9.** Top Management Takeover: Once user testing is concluded and the prototype transforms into a functional ready-to-use system, next top management takes over, conducts comprehensive testing and evaluation of entire new system components and functionalities against the project sign off document
**2.** Develop your organizational digital strategy preceding the digital transformation journey. This would guide your choices for both present and future digital transformation decisions: Do we design and implement or do we adopt? Or do we outsource to third party providers	**6**. Ideation/Brainstorming Session: Once the problem had been defined and stakeholders agreed, next is that the technical team would embark on brainstorming session to arrive at all possible solution options and alternatives and arrives at best intuitive solution to address these pain-points; also called the user needs	**10. **If your hospital digital transformation is by way of adoption of Vendor/Commercial off the Shelf Projects then:a. Involve all relevant stakeholders including the usersb. Ensure system alignment with clinical workflowc. Ensure system compliance with legal and regulatory frameworkd. Ensure End User involvement in system’s testing and evaluatione. Develop a comprehensive User training programmef. Avail after sales support till user are accustomed to new system
**3.** Conduct Needs Assessment a. Assess care facility and stakeholders readiness to embrace digital transformation drive, maybe using a digital readiness assessment survey to ensure all or major digital transformation enablers are in place: availability of sufficient digital skillset amongst staff population, presence of supporting infrastructure like power supply, internet connectivity backbone, evidence of strong commitment and consistency of top management support and political will, required funding, staff motivation, bureaucratic bottlenecks, maintenance culture, early involvement of stakeholders right from project inception, across planning stages etc. b. Conduct focus group interviews (either or both structured and open) c. Evaluate of existing infrastructure/system d. Observe existing workflow and visualize end-user journey map	**7**. System Modelling: Once a choice had been made from brainstorming session, next step is to model the system using Unified Modelling Language. This helps the developers be able to conceptualize the system functionalities and logical flow across the various entities of the system	**11**. New system deployment begins: Once the top management is done with the system testing, next step is the roll out of hospital-wide hardware and software infrastructure to support the new system set up; including all necessary installations **12**. Training for the new system use: After the new system deployment a comprehensive training programme should be developed to support effective use of new system
**4.** Appoint a hospital-wide digital transformation project team with stakeholders well represented	**8**. Prototyping: After the system had been modelled using UML diagrams next is to develop and test/evaluate the prototype of the proposed solution amongst the end users.a. Once your prototype is ready next is to conduct first prototype testing and evaluation by end users.b. After first iteration, improve your prototype with user feedback and go back for a second time prototype testing/ evaluation	**13**. The digital transformation cycle ends (project closure): Once all concerned staff population had been adequately trained and system use began, then the digital transformation journey can then be officially declared to have come to end. Now is time to take stock of the entire digital transformation journey, the challenges and the lessons.

To operationalize the proposed framework within the broader TUMERIC research program, an iterative human-centered implementation process grounded in Agile development and participatory design principles was employed. The process incorporated organizational readiness assessment, workflow analysis, stakeholder engagement, iterative prototyping, usability evaluation, deployment planning, and post-deployment training activities. The stepwise implementation pathway applied during the study is summarized in Table 2 below.

Meanwhile Fig. 9 shows the sequence diagram of the empirically derived healthcare digital transformation implementation framework.

Again Fig. 10 represents the Mind Map of the proposed empirically derived healthcare digital transformation implementation framework.

**Fig. 11:**
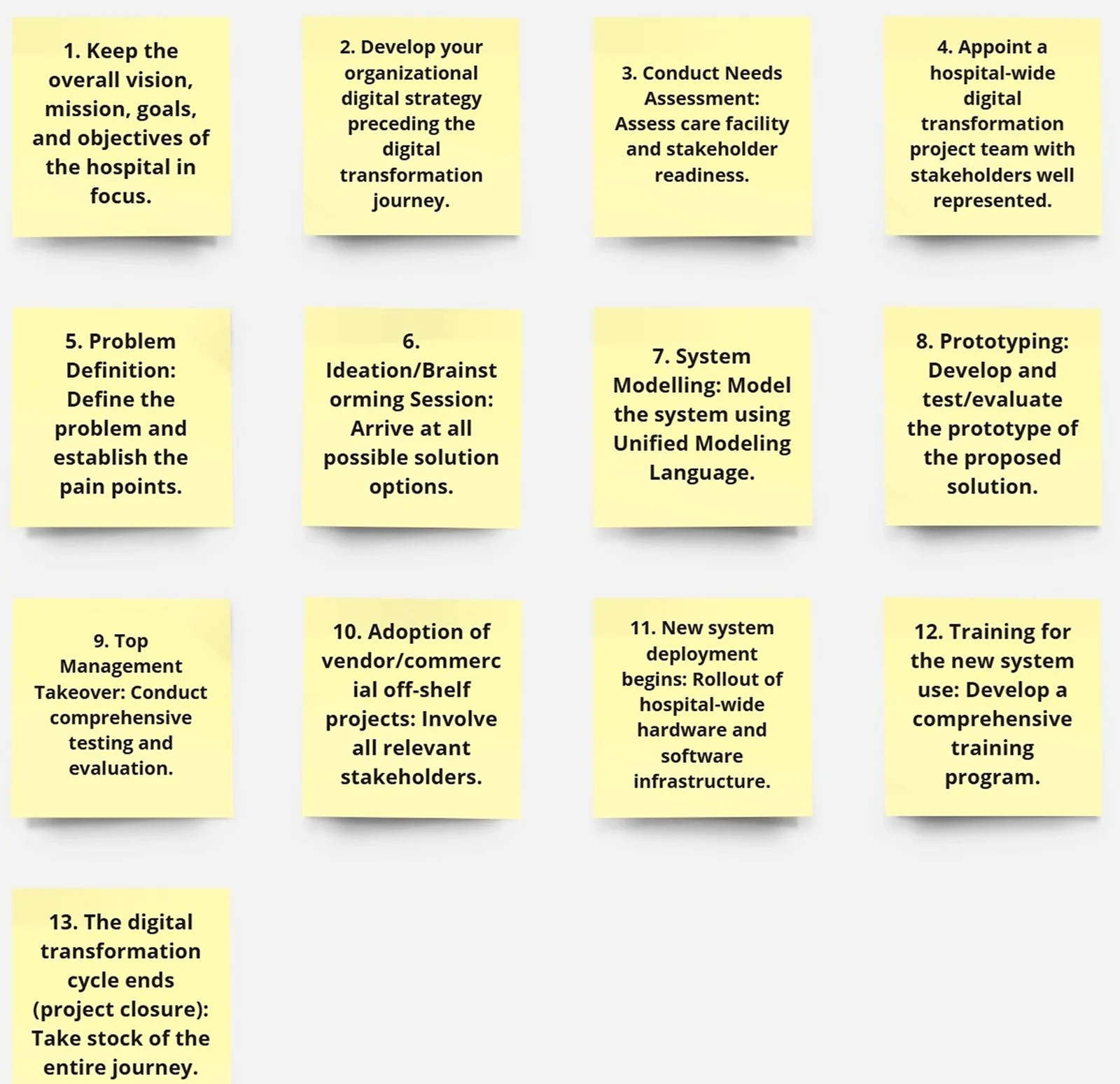
Sticky notes for high-level illustration of the empirically derived healthcare digital transformation implementation framework

Again, the Fig. 11 presents the high-level illustration of the empirically derived healthcare digital transformation implementation framework.

## Discussion

This study investigated the challenges, readiness factors, user experiences, and implementation barriers influencing healthcare digital transformation across tertiary healthcare institutions in southeastern Nigeria and subsequently developed an evidence-based digital transformation framework grounded in agile human-centered software engineering principles. The findings demonstrated moderate but variable organizational readiness for digital transformation across the participating hospitals, with many healthcare professionals expressing support for future digital initiatives. The study also identified substantial usability and workflow-related limitations within existing digital systems, including poor interface design, documentation burden, workflow disruption, and inadequate alignment with clinical practices. Qualitative findings further revealed recurring concerns relating to infrastructural limitations, insufficient stakeholder involvement, resistance to change, digital literacy gaps, and weak organizational support structures. Through participatory design, iterative prototyping, and usability evaluations, the developed TUMERIC cloud- and voice-enabled healthcare support system demonstrated high levels of perceived usability and user acceptance among clinicians and patients. Collectively, these findings informed the development of the proposed evidence-based healthcare digital transformation framework tailored to the realities of tertiary healthcare institutions within developing and resource-constrained environments.

This segment discussed the various components and dimensions of the empirically derived healthcare digital transformation implementation framework. It should also be understood that these various components are iterative and can be revisited throughout the implementation cycles until the overall goals are fully actualized.

1. **Keep the overall mission**,** vision**,** and social and business goals and objectives in focus**: Engage with the senior leadership of the healthcare organization to gain a comprehensive understanding of the both the long-term and short-term objectives, as well as the overarching vision and mission of the institution, in its capacity as both a commercial entity and a social establishment. These objectives are frequently articulated within the organizational vision and mission declarations. Each instance of technology adoption and implementation must align with the overarching business objectives of the institution. It is imperative to recognize that digital transformation is fundamentally aimed at facilitating business transformation, thereby altering the manner in which organizations engage with critical stakeholders, including clients, customers, and the internal workforce, particularly through the enhancement of user experience. Furthermore, it is essential to acknowledge that individual units within the organization may possess distinct goals and business ambitions. It is crucial to identify and integrate these aspirations into your digital transformation strategy, especially as they pertain to each specific department and unit.
2. **Digital Strategy**: Examine whether there exists an operational digital strategy within the healthcare facility. Should such a strategy be currently implemented, it is plausible that it may be antiquated and necessitate a comprehensive review. The responsibility rests with the Chief Digital Officer (CDO) of the healthcare institution to develop the digital strategy and ensure its alignment with the organization’s overarching and immediate business objectives. It may be prudent to seek external expertise, given their extensive experience in initiatives pertaining to digital transformation.

When formulating a digital strategy for your healthcare institution, it is imperative that the resultant document addresses certain pivotal inquiries. This assertion is grounded in our empirical observations derived from extensive field research, thus reinforcing its evidence-based nature. Below are the essential questions that any digital strategy must comprehensively address:

- In what manner can digital technology or the acquisition of technological resources facilitate our healthcare institution’s ability to maintain global competitiveness and enhance the experiences of our clients and customers, particularly with respect to the efficiency of service delivery, cost-effectiveness, and overall user experience?
- In what ways can digital technology or the procurement of technological assets aid our healthcare institution in refining our internal operations and enhancing the experiences of our staff, especially regarding the swiftness of service delivery, operational efficiency, effectiveness, and prudent cost management?
- How can digital technology or technological acquisitions empower our healthcare institution to either disrupt or establish a dominant presence within the current market landscape?
- In what ways can digital technology or the acquisition of technological resources enable our healthcare institution to broaden our service offerings, explore new market opportunities, and enhance our growth potential?
- Which specific departments, units, or service offerings within our organization would necessitate particular types of technological integration in order to achieve our social and business aspirations, visions, and objectives?
- What specific competencies, including digital skills, must we cultivate or recruit in order to effectively implement our digital strategy?
- What types of collaborations (including digital partnerships, such as alliances with Healthcare Systems Development Companies) should our healthcare institution pursue to ensure the successful execution of our digital strategy?
- Are there any applicable state legal and regulatory frameworks that our digital strategy must adhere to?
- What digital roadmap (a systematic, stepwise approach) or playbook must our healthcare institution follow to implement our digital strategy (for instance, employing design thinking, service design, and other human-centered development frameworks that may facilitate a structured approach to the successful implementation of our digital strategy)?
- Are there any potential risks (including digital risks) that could impede the execution of a sustainable digital strategy for our healthcare institution?

It is imperative at this stage to articulate that the implementation of digital strategy through digital initiatives and endeavors is also in accordance with any recognized project management framework that facilitates the execution of various types of projects. Consequently, all significant deliverables outlined in the standard PMI PMBoK, for example, are equally applicable during the execution of digital strategy programs and projects.

3. **Needs Assessment**: Conduct an exhaustive digital needs assessment at the healthcare facility to investigate the healthcare and ancillary services that necessitate digital intervention. This methodology may encompass: Digital readiness assessment survey: This would assess the psychological and technical preparedness of stakeholders to adopt the forthcoming technological intervention. The implementation of this survey should span the entirety of the units and departments that are being primed for transformation. The perceptions and impressions of all clinical stakeholders were scrutinized to identify potential obstacles to a successful digital transformation initiative. It is crucial to administer these questionnaires offline (where practicable, particularly in developing nations where individuals may possess limited technological proficiency and the costs associated with internet access are relatively elevated, which diminishes stakeholders’ willingness to participate in an online survey). Offline questionnaires in this context would therefore facilitate researchers in following up and ensuring completion until personnel have submitted their responses and the completed questionnaires are collected.

- **Interviews (both structured and open)**: This represents another significant method for conducting a needs assessment. This can also be executed at the focus group level. Departments and Units could, accordingly, facilitate straightforward and candid interactive sessions. Stakeholders would be anticipated to articulate their concerns and reservations regarding the new project initiative.
- **Evaluation of existing infrastructure and systems**: As an integral component of the needs assessment process, there exists a necessity to appraise the current infrastructure, including technological frameworks, alongside their operational effectiveness. The design and user experience associated with those systems warrant investigation and evaluation. Each Unit and Department must be capable of delineating all pain points, challenges, desires, and expectations pertaining to the previous system. This exercise can be coordinated unit by unit and Department by Department by their designated digital champions. Questionnaires and interviews can bolster this phase.
- **Observation and customer journey map**: In furtherance of the needs assessment discourse, it is frequently recommended that the system implementation team (within a human-centred computing framework) allocate time to visit the implementation site to conduct a meticulous examination of the current operational state prior to the initiation of the change management process. Observation serves to unearth the pain points within the extant system. It elucidates the frustrations experienced by the staff population, as one can often witness the difficulties they encounter and the grievances they articulate. Implementing a change management initiative, particularly within the realm of digital transformation, necessitates considerable time and commitment to realize success. Observation would also assist the development team in conceptualizing the clinical workflow of the staff population to construct systems that are congruent with the prevailing healthcare workflow in the facility under examination. Furthermore, observation becomes imperative in aiding the team to formulate a patient journey map that is centered around the end-user. The patient journey map delineates every point of patient data capture and the associated functionalities that are to be incorporated within the digital transformation projects.

4. **Appointment of the hospital-wide Digital Transformation project team**: It is imperative to establish a comprehensive digital transformation project team within the hospital, which shall include an appointed Digital Champion and potentially a deputy Digital Champion (to ensure uninterrupted availability) representing each Unit or Department of the tertiary or university hospital undergoing the transformation process. The primary responsibility of the Digital Champion is to inspire and encourage their respective units to actively participate in the digital transformation initiative. Furthermore, it is incumbent upon the Champions to ensure that all concerns and anticipations of their distinct Units and Departments are articulated during the implementation phase. These Digital Champions shall also be tasked with bringing to light any early prototype deficiencies pertinent to their respective units at the earliest opportunity. Digital Champions should possess a high degree of technological proficiency and, notably, should be collaborative individuals exhibiting additional leadership attributes. This digital transformation project team shall also encompass members from the ICT department of the hospital, a representative from top management, the hospital’s Chief Digital Officer, and Digital Transformation Consultants (in instances where this transformation journey has been outsourced to a Healthcare Systems Development organization). It is noteworthy that several of the Digital Champions may concurrently represent both clinical and non-clinical stakeholders, who are themselves likely end users.
5. **Problem definition**: Based on the outcomes derived from the digital readiness assessment survey, the conducted interviews, the appraisal of existing infrastructure and systems, as well as the observations and patient journey mapping, the digital transformation consultant and their team spearheading the campaign will delineate the challenges, pain points, and overarching expectations of end users. The findings from this stage shall be presented during a hospital-wide digital transformation project team meeting for ratification, thereby signaling the formal approval and initiation of the hospital-wide digital transformation program or project.
6. **Ideation/Brainstorming Session**: During the most recent stakeholder meeting, the problems and challenges associated with the transformation were unanimously acknowledged. The conducted interviews, questionnaires, observations, and end-user journey maps facilitated the identification of these challenges. These pain points have now been systematically extracted and accepted. A pivotal meeting is forthcoming—a brainstorming session among the technical team to assess the available solution options. The technical team may comprise individuals with multidisciplinary expertise; personnel from human-centered computing disciplines would be particularly advantageous. This team is adept at executing user-centered digital projects, including those pertaining to healthcare transformation. At this juncture, they are convening to evaluate the solution options in alignment with the overarching vision, mission, and objectives of the health facility. They must exhibit intuition and empathy, placing themselves in the perspective of the end users and endeavoring to experience their challenges. Upon selecting the optimal approach, the subsequent step involves the development of a prototype. The technical team may consist of Software Engineers, Systems Analysts, Digital Transformation Consultants, Human-Centered Computing specialists, among others.
7. **System Modelling**: The current juncture necessitates the initiation of a comprehensive model of the system artefact. This endeavour may be facilitated through the application of the Unified Modelling Language (UML). Such a framework would assist developers in visualizing the internal logic and the distribution of information across diverse segments and entities of the proposed digital healthcare intervention system. Developers rely significantly on UML; it serves as an essential tool for them to conceptualize the operational functionalities of the proposed system. The Unified Modelling Language encompasses various tools, including Use Case Diagrams, Class Diagrams, Data Flow Diagrams, Entity Relationship Diagrams, and other algorithms pertinent to the modeling of healthcare intervention systems. However, the end user, within the paradigm of Human-Centred Computing, demands a different deliverable—a prototype that provides a concrete representation of the proposed system. Both sets of stakeholders play a crucial role in determining the overall outcome of the system, necessitating that both parties are sufficiently engaged through the utilization of appropriate tools. This progression leads us to the subsequent phase of prototyping.
8. **Prototyping**: The ensuing phase involves the design and development of the inaugural functional prototype. The Digital Transformation Consultant and the development team must remain cognizant of the fact that users of these technologies in developing and resource-constrained economies frequently lack a clear understanding of how the system they require will ultimately manifest. Consequently, they often depend on the intuition and expertise of the Digital Transformation Consultants, along with their team of User Experience Designers or systems developers. Clinicians operating within these economies are typically constrained by demanding schedules and a considerable patient load, rendering them unable to engage with static paper mockup representations. They require functional systems to be presented to them, at which point they can optimally contribute to the system development process by requesting modifications in interface design, advocating for the inclusion of specific functionalities, and expressing concerns regarding the safety and security implications inherent in the system design.

Moreover, in light of the clinicians’ demanding schedules and those of associated staff, there exists limited capacity for excessive iterations and meetings. Therefore, the digital transformation team, comprising Digital Transformation Consultants, UI/UX specialists, Systems Analysts, and Developers, must demonstrate a high degree of intuition in devising solutions that align with the psychological needs of potential users while minimizing the number of iterations. To expedite the early release of an interactive prototype for user assessment, Systems Analysts, who may also be integral members of the Digital Transformation team, should commence preliminary modelling of the proposed system utilizing UML tools as previously outlined, while Developers and UI/UX designers collaborate to create interactive prototypes that reflect the psychology and emotional responses of potential users, particularly in adherence to all usability standards concerning simplicity, ease-of-use, learnability, and the fulfillment of all proposed functional requirements. Indeed, drawing from our field experience and in consideration of the hectic healthcare milieu characteristic of developing economies where clinics are frequented by large patient populations, it is advisable that system development iterations, which involve substantial user participation, should be limited to no more than two.

### First prototype user testing and evaluation

In the initial iteration of prototype assessment, users engage in a comprehensive examination of the prototype, providing substantial insights for modifications to the presented model. These user evaluations are conducted at the unit or departmental levels, contingent upon the various dimensions of the system currently under development. Each department or unit is exclusively exposed to the specific dimension or segment of the system that directly pertains to their operational activities. It warrants acknowledgment that this procedure is time-intensive, and the overall success or failure of the entire system implementation is contingent upon the integration of the ultimate end-users throughout the system development value chain, adhering to the user participatory development or co-creation paradigm. Thus, the first user prototype evaluation constitutes a critical component of the user-centric system development process.

During this initial phase of the prototype evaluation, users are encouraged to articulate their perceptions regarding the execution of certain system functionalities. They are prompted to express their preferences for modifications or the incorporation of additional features and functionalities, as well as the elimination of existing ones. Users may also raise issues concerning any discrepancies within the system interface design; they could notify the development team of challenges related to dialogue boxes and notifications pertaining to errors and successes. It is imperative that users are afforded the opportunity to freely convey their impressions and viewpoints regarding the system prototype. Employing ethnographic protocols, direct observations, and interviews are effective methodologies at this stage of prototype evaluation. This approach enables the development team to promptly address users’ feedback.

Subsequently, the development team revisits the feedback gathered from users, including their opinions, impressions, grievances, and commendations, and begins to reconcile these modifications with the project sign-off document, which delineates the expectations of all stakeholders concerning the proposed project.

### Second prototype user testing and evaluation

In this subsequent phase of prototype evaluation, users seek to ascertain whether all requested modifications have been comprehensively integrated. During this stage, they commence familiarizing themselves with the new system and evaluating its consistency with the clinical workflow of the care facility. It remains possible for users to identify additional adjustments to the system; realizing all user-suggested changes would facilitate their full ownership of the system as co-creators. At this juncture, users would also juxtapose the enhanced system or prototype against the project sign-off document to verify that all stakeholders’ expectations have been satisfactorily fulfilled. It is important to note that this second prototype user evaluation will continue to occur at the unit or departmental level, with each unit or department being exposed solely to the dimensions of the system that influence their clinical operational workflow.

Moreover, the developers will once again gather the final user input and reflect accordingly; in instances where user complaints are minimal at this stage, subsequent improvements may not necessitate another extensive user evaluation. A limited number of user representatives would likely find this acceptable.

9. **Top Management Takeover**: Subsequent to the completion of user co-creation through evaluations of user prototypes, which were also overseen by a representative from Top Management, the subsequent phase involves the transition to the conclusive review and evaluation conducted by management. During this phase, it is anticipated that the management representative will collaborate with the Information and Communication Technology (ICT) Department of the healthcare facility to meticulously assess the full range of functionalities of the system; the safety and security protocols will be scrutinized in accordance with all pertinent governmental regulations and standards. Upon confirming that all protocols and standards have been satisfied, the rollout and deployment of the system, in partnership with the ICT Department, will commence.
10. **Healthcare Digital Transformation through the adoption of vendor or commercially available off-the-shelf projects**: There are instances in which healthcare institutions may opt not to develop their digital intervention projects from the ground up; rather, they may choose to implement systems that have already been established by healthcare systems development firms. Should this methodology align with the circumstances surrounding your healthcare institution, it is imperative to consider several critical factors: Involve all relevant stakeholders, including the users: It is essential to engage all pertinent stakeholders, particularly the end users, in the process. Indeed, the committee established by management to oversee such acquisitions must consist of representatives from all final end users, the ICT Department, and the digital champions within various units or departments. Their involvement is crucial in the thorough testing and evaluation of the system’s demo version, encompassing its functionalities, interface and interaction designs, as well as its capacity to support the existing clinical workflow. This evaluation and testing should be conducted repeatedly until a broad consensus of user acceptance is attained.

- **Alignment with clinical workflow**: Any selection of a digital intervention project must consider whether its design is congruent with the existing clinical workflow. Generally, users exhibit a preference for systems that maintain the workflow to which they have become accustomed over an extended period.
- **Compliance with the legal and regulatory framework**: For any digital intervention to be deemed suitable for acquisition, it must adhere to the legal and regulatory framework established by the State. Consequently, all safety and security features of the system must conform to the standards set forth by the State. For example, digital health intervention systems and projects are required to comply with National and Regional Data Protection Acts and Regulations.
- **User Involvement in the System’s Testing and Evaluation**: Furthermore, representatives of the end users should undertake both independent and collective evaluations of the system’s usability, and an assessment of their user experience (UX) should be clearly documented.
- **User Training**: Following the selection of any system, a comprehensive training program should be meticulously designed and executed.
- **After-Sales Support**: The sales contract should encompass provisions for after-sales support until the healthcare facility has fully acclimated to the use of the newly implemented system.

11. **A new system deployment commences**: At this juncture, there will be a comprehensive rollout of all essential infrastructures (both hardware and software) to facilitate the new system. At this point, the digital champions within all Units and Departments will ensure that all prospective end users are engaged during this phase. A novel digital operational culture is poised to initiate.
12. **Training for the utilization of the new system**: Following the deployment of the new system, staff training will commence to guarantee the effective and efficient application of the new system. Subsequently, the heads of departments and units will be granted the rights and privileges necessary to enroll their respective staff members. These administrators will also receive training on fundamental troubleshooting techniques and strategies to navigate any challenges encountered while utilizing the new system. Following this phase, a comprehensive training program will be conducted for the staff population who will be responsible for various aspects of the new system across different units and departments. This training is anticipated to be thoroughly detailed and exhaustive.
13. **The project implementation cycle concludes (project closure)**: At this stage, the new system is fully operational across the designated units and departments. The staff population is gradually acclimating to a new modus operandi, a digital culture, which contrasts with the previous manual processes or, in certain instances, outdated digital systems that were inadequately implemented, leading to difficulties for clinicians in their usage. It is now imperative to evaluate the challenges and success narratives encountered throughout the digital transformation process.

### Potential applicability of the evidence-based digital transformation framework to other developing economies

Now, the proposed evidence-based digital transformation framework offers a structured approach to enhancing healthcare delivery through digital solutions, particularly in resource-constrained environments. As many developing economies faced similar challenges in their healthcare systems, understanding the commonalities and differences in their contexts can broaden the framework’s relevance and applicability. This discussion explores these aspects, highlighting how the framework can be adapted to meet the unique needs of various developing economies while maintaining its core principles.

### Commonalities between other developing economies and Nigeria vis-à-vis the framework

Developing economies often operate under tight budgets, limiting their ability to invest in comprehensive digital solutions, which necessitates a focus on prioritizing essential digital tools that deliver maximum impact with minimal investment. Many healthcare systems in these regions struggle with inadequate technological infrastructure, including unreliable internet access and outdated hardware, hindering effective implementation. Additionally, engaging a diverse range of stakeholders is critical for success, as healthcare providers, patients, and administrative staff may have differing perspectives and needs. The framework’s emphasis on participatory design ensures that all voices are heard, fostering a sense of ownership and increasing the likelihood of successful adoption. Furthermore, the integration of digital tools into existing clinical workflows is essential for minimizing disruption and enhancing efficiency, ensuring that new technologies complement established practices.

### Differences between other developing economies and Nigeria vis-à-vis the framework

Cultural attitudes towards technology and healthcare can significantly influence the acceptance of digital solutions, with some regions exhibiting a strong preference for traditional methods that may lead to resistance against new technologies. Each country also has its own healthcare regulations and policies that can impact the implementation of digital transformation initiatives, necessitating flexibility in the framework to accommodate legal requirements. Economic stability and growth rates vary widely among developing economies, affecting the availability of funding for digital initiatives, which may require prioritizing low-cost, high-impact interventions. Additionally, the level of technological advancement and digital literacy can differ significantly, with some countries needing foundational training and support before adopting advanced digital solutions, highlighting the importance of assessing local technological readiness.

### Summarized comparative analysis of key findings from empirical phases with the proposed framework

Mean Table 3 provides critical insights into patient evaluations of the usability of the developed digital healthcare support system. The high mean scores indicate that patients found the system user-friendly and effective in meeting their needs, which is essential for ensuring successful adoption in healthcare settings. This positive feedback aligns with the framework’s emphasis on human-centered design principles, highlighting the importance of involving end-users in the development process to create solutions that are intuitive and accessible. The findings suggest that when patients perceive a digital system as usable, they are more likely to engage with it, thereby enhancing the overall quality of care.

**Table 3:** Patients’ usability evaluation of the implemented digital healthcare support system using an adapted usability evaluation questionnaire informed by System Usability Scale (SUS) principles (No of respondents: 20 Patients)

S/No	Questionnaire Item	Mean	Standard Deviation	Sum	No. Valid	Missing Values
1.	I think that I would like to use the Electronic Health Record System frequently	**4.25**	0.910	85	20	0
2.	I found the Electronic Health Record System to be simple	**4.20**	0.616	84	20	0
3.	I thought the Electronic Health Record was easy to use	**4.10**	0.641	82	20	0
4.	I think that I could use the Electronic Health Record System without the support of a technical person	**4.40**	0.754	88	20	0
5.	I found the various functions in the Electronic Health Record System were well integrated	**4.15**	0.587	83	20	0
6.	I thought there was a lot of consistency in the Electronic Health Record System	**4.25**	0.550	85	20	0
7.	I would imagine that most people would learn to use the Electronic Health Record System very quickly	**4.15**	0.745	83	20	0
8.	I found the Electronic Health Records System very intuitive	**4.30**	0.657	86	20	0
9.	I felt very confident using the Electronic Health Record System	**4.05**	0.605	81	20	0
10.	I could use the Electronic Health Record System without having to learn something new	**3.70**	0.923	74	20	0

Furthermore Table 4 presents the results of the readiness assessment research, which evaluates the preparedness of healthcare units to implement digital solutions. The data indicates that a significant proportion of healthcare staff feels ready to adopt new technologies, reflecting a favorable organizational climate for digital transformation. This readiness is crucial for the successful implementation of the proposed framework, as it underscores the importance of assessing and fostering a supportive environment for change. The findings suggest that when healthcare institutions prioritize readiness assessments, they can better align their digital transformation efforts with the capabilities and attitudes of their staff, ultimately leading to more effective and sustainable adoption of digital tools.

**Table 4:** Result of doctors perception of the digital readiness of UNTH to implement healthcare digital transformation programs and projects number of respondents: Responses of 73 Doctors

Latent Variables	Questionnaire Item	Strongly Disagree	Disagree	Neither Agree nor Disagree	Agree	Strongly Agree	Total %ages
Vision Clarity (VC)”	I believe there are legitimate reasons for us to introduce a computer-based system in our hospital	3.0%	0%	12.1%	33.3%	51.5%	100%
	We need tools to improve the way we work around here	0%	0%	6.1%	36.4%	57.6%	100%
	There a number of rational reasons for the deployment of EHR in our hospital	0%	0%	12.1%	48.5%	39.4%	100%
	A Computer-based system is needed to improve our clinical processes	0%	3.0%	12.1%	48.5%	36.4%	100%
Change Appropriateness (CA)	I think that staff in our unit and our hospital at large will benefit from the use of Electronic Health Record	0%	0%	6.1%	48.5%	45.5%	100%
	The deployment of an EHR will contribute to hospital overall performance	0%	3.0%	12.1%	36.4%	48.5%	100%
	The deployment of an EHR would surely match the priorities of our hospital	0%	0%	27.3%	57.6%	15.2%	100%
	The implementation of EHR will prove to be a best decision for our hospital	0%	0%	30.3%	48.5%	21.2%	100%
Change Efficacy (CE)	I know staff outside our hospital and in other similar hospitals in Nigeria who had successful experiences with an EHR	3.0%	12.1%	12.1%	45.5%	27.3%	100%
	An EHR has been successfully deployed in other hospitals similar to our hospital	3.0%	6.1%	21.2%	54.5%	15.2%	100%
	An EHR has received positive reviews in the press (e.g. newspapers, magazines, seminars, etc.)	0%	12.1%	42.4%	39.4%	6.1%	100%
	I believe government policy on EHR would represent a driving force for the deployment of EHR in our hospital	0%	6.1%	33.3%	42.4%	18.2%	100%
Top-management support (TMS)	The Administrators in our hospital are committed to the deployment of EHR	0%	0%	30.3%	60.6%	9.1%	100%
	The Administrators in our hospital had stressed the importance of this change	0%	0%	33.3%	48.5%	18.2%	100%
	The (hospital) administrators have sent clear message that the deployment of EHR will occur in our hospital	0%	0%	27.3%	48.5%	24.2%	100%
	Staff have been encouraged to embrace any upcoming deployment of EHR	0%	0%	18.2%	66.7%	15.2%	100%
Presence of an Effective Champion (C)	There is a key staff/champion who would be able to push EHR projects over and around implementation hurdles.	0%	24.2%	42.4%	30.3%	3.0%	100%
	Any proposed EHR project would readily find a credible and trustworthy staff championing its implementation	0%	21.2%	36.4	42.4%	0%	100%
	There is a key staff/Champion who actively promotes the deployment of EHR in our hospital.	0%	15.2%	39.4%	42.4%	3.0%	100%
Organizational History of Change (OHC)	Our hospital had successfully implemented other technological changes in recent years.	3.0%	18.2%	45.5%	33.3%	0%	100%
	Staffs in our hospital have had negative experiences with technological projects in the past.	0%	6.1%	54.5%	36.4%	3.0%	100%
	Our hospital is usually successful when it undertakes all types of changes.	0%	24.2%	39.4%	30.3%	6.1%	100%
	Information Technology initiatives have been encouraged and are common practices in our hospital	0%	12.1%	21.2%	57.6%	9.1%	100%
Organizational Conflicts and Politics (OCP)	Mutual trust and cooperation among staff in our unit is strong.	3.0%	9.1%	42.4%	42.4%	3.0%	100%
	Recent attempts to change the way we work in our hospital have been hindered by political forces or conditions	3.0%	27.3%	45.5%	21.2%	3.0%	100%
	The climate in our hospital is mainly characterized by conflicts and disputes	12.1%	45.5%	18.2%	15.2%	9.1%	100%
	Staff frustration is often common in our hospital	3.0%	18.2%	18.2%	42.4%	18.2%	100%
Organizational Flexibility (OF)	Our hospital is structured to all superiors make changes quickly	0%	24.2%	42.4%	33.3%	0%	100%
	It is easy to change to change procedures in our hospital to meet new conditions	3.0%	15.2%	48.5%	33.3%	0%	100%
	Getting anything changed in our hospital is a long, time-consuming process	0%	18.2%	36.4%	30.3%	15.2%	100%
	Policies and Procedures in our hospital allow us to take on new challenges effectively	9.1%	9.1%	54.5%	27.3%	0%	100%
Collective Self-Efficacy (CSE)	All staff in our unit are highly computer literate.	12.1%	33.3%	42.4%	9.1%	3.0%	100%
	It won’t take a long time before staffs in our unit feel comfortable using any newly implemented EHR-System project.	3.0%	18.2%	30.3%	48.5%	0%	100%
	Using computer effectively is no problem for the staff in our hospital.	9.1%	9.1%	39.4%	39.4%	3.0%	100%
	In general staff our unit have low computer skills.	3.0%	36.4%	39.4%	21.2%	0%	100%
Organizational Readiness (OR)	I believe EHR can be successfully implemented in our unit.	0%	3.0%	3.0%	66.7%	27.3%	100%
	I wish Administrators should delay the deployment of EHR in our hospital	15.2%	63.6%	15.2%	6.1%	0%	100%
	The deployment of an EHR-System in our hospital is timely.	3.0%	9.1%	24.2%	51.5%	12.1%	100%
	Our unit is ready to take on this technological change	0%	3.0%	33.3%	54.5%	9.1%	100%

### Limitations of the proposed framework

Despite the important contributions of this research, certain limitations should be acknowledged. The successful implementation of the proposed framework remains dependent on several external institutional and infrastructural factors, including stable electricity supply, broadband connectivity, technical support capacity, digital literacy, organizational leadership commitment, and governmental policy support. These structural limitations remain significant challenges within many developing and resource-constrained healthcare environments and may therefore influence implementation outcomes differently across institutions.

In addition, although the study integrated both quantitative and qualitative methodologies, some qualitative findings were based on self-reported experiences and perceptions from clinicians and stakeholders, which may reflect variations in individual experiences and institutional contexts. Nevertheless, the use of mixed-methods triangulation, participatory stakeholder engagement, direct observational methods, and iterative usability evaluations helped strengthen the credibility and contextual reliability of the findings.

Despite these limitations, the proposed framework provides an important empirically grounded contribution toward understanding and supporting healthcare digital transformation within tertiary healthcare institutions in developing economies.

## Conclusion

This framework has been meticulously designed to reflect global best practices, incorporating the principles of agile methodologies and design thinking. It is inherently human-centric and end-user-focused, particularly within a participatory context. Moreover, it empathetically addresses the prevailing challenges faced by healthcare institutions in developing and resource-constrained environments. This report outlines a step-by-step approach—many aspects of which the researchers have practically implemented over the years with rewarding outcomes - while investigating factors that may support more effective and sustainable healthcare digital transformation programs and projects. The focus is particularly on the complex tertiary healthcare landscape of university teaching hospitals in Nigeria. The report presents these dimensions in a simplified manner, ensuring clarity while capturing the overall essence of any applicable healthcare institution. It also emphasizes the involvement of all relevant stakeholders within a participatory systems engineering framework. The framework is informed by the researchers’ direct experience working within the same healthcare environment in a developing country. Additionally, it incorporates crucial policy and regulatory considerations that must be factored in when deploying healthcare technologies. Empirical findings from this study showed that patients rated the usability of the digital healthcare support system implemented in this research journey with high mean scores, demonstrating high usability. These findings suggest that agile and human-centered systems engineering practices may have contributed positively to usability perceptions within the study context. More results showed that over 70% of healthcare workers thought their facility was ready to embrace new digital initiatives. These outcomes, as expected, would guide further leadership decisions on strategy for future digital transformation initiatives. It is important to note that these outcomes underscore the relevance of the proposed digital transformation framework, which was built upon this evidence and design philosophy. It is, therefore, anticipated that this evidence-based framework will serve as a practical guide for healthcare decision-makers, researchers, and digital transformation consultants exploring opportunities in the sub-Saharan African market. Additionally, it highlights key considerations essential for developing digital strategies in resource-constrained contexts.

## Supplementary Information

Below is the link to the electronic supplementary material.

Supplementary Material 1

Supplementary Material 2

Supplementary Material 3

## Data Availability

All data supporting the findings of this study are available within the paper and its Supplementary Information.
