# Supplementary material for: The consultant’s guide: evidence-based digital transformation framework for healthcare institutions in developing and resource-constrained economies
Source: BMC Med Inform Decis Mak. 2026 Jul 16;26:405. doi: 10.1186/s12911-026-03713-9 (PMC13659990; doi:10.1186/s12911-026-03713-9)
Supplement: Supplementary file 1 [file 12911_2026_3713_MOESM1_ESM.pdf]

Open Ended Interview Questions to Assess the Opinions of Clinicians on Challenges and Limitations to Adoption of Healthcare Digital Intervention Programmes and Projects across Tertiary/University Teaching Hospitals in Developing Countries

1. Do you think your hospital is Ready or Not Ready to implement an EHR? Please tick Ready or Not Ready as applicable to your hospital, and then give reasons for your answer in a short comment.
2. If there had been any computer-based Electronic Health Record project attempted in the past in your hospital, to what extent do you think it succeeded, and was it able to support doctors and nurses to store and retrieve patients' health records effectively?
3. If past Electronic Health Record System implementation in your hospital failed or performed below expectations and was later abandoned by the doctors or the nurses or both, who later returned to the paper mode of storing patients' health information, what do you think caused such failure? What were the complaints made by the doctors and nurses who used them (if any)?
4. What challenges or limitations do you think could hamper future implementations of computer and internet-based digital transformation programmes and projects in your hospital if not addressed?
