# Supplementary material for: The consultant’s guide: evidence-based digital transformation framework for healthcare institutions in developing and resource-constrained economies
Source: BMC Med Inform Decis Mak. 2026 Jul 16;26:405. doi: 10.1186/s12911-026-03713-9 (PMC13659990; doi:10.1186/s12911-026-03713-9)
Supplement: Supplementary file 2 [file 12911_2026_3713_MOESM2_ESM.pdf]

Healthcare Digital Transformation: A Survey to Evaluate Use-based Challenges Usability and General User Experience of Clinical Stakeholders Working with Existing EHR-Systems at the Tertiary/University Hospitals under Study

Short questions designed to help us identify any challenges you are facing with existing or recent patient Electronic Health Records clerking system currently or previously used by you as a Doctor, Nurse and Medical Records Officer in this Hospital. This would help us identify areas to improve the performance of these systems.

Participant chosen initials (Please do not use initials that could identify you): _____________Date: ____________________

Your responses would be strictly anonymous and confidential but would also help us know the improvements the electronic health record clerking system requires.

PART A

1. **What is your age** (Years) __________

2. **Please indicate your gender**: Male ☐ Female ☐

3. **How long have been working at this hospital**? (Years): __________

4. **What is your highest level of education**: High school ☐ Diploma ☐ Bachelors ☐ Masters ☐ PhD/Fellowship ☐

5. **Please indicate your job title**: Doctor ☐ Nurse ☐ Health Record Officer ☐

6. **Please could you let us know if your daily work involves direct patient care?** Yes ☐ No ☐

7. **Could you state how often you used a computer?** Daily ☐ A few times a week ☐ A few times a month ☐ A few times a year ☐ Never ☐

8. **On a scale of 1 to 5 please rate your computer skills, where 1**= Basic computer skills (need help with internet and email or office applications), **5**= Proficient

Please tick as you think appropriate, your level of computer skill on the scale of 1 to 5 below:

Basic 1 — 2 — 3 — 4 — 5 Proficient

9. **Do you have any experience using electronic medical records (EHR) systems?** Yes ☐ No ☐

10. **Have you ever received any training on the use of EHRs?** Yes ☐ No ☐

PART B: This section evaluates the usability and your experience using the new clerking system.

1. I feel the interface design of this electronic health records clerking system is crowded with some unnecessary information there are never used.

Strongly Agree	Agree	Neither Agree nor Disagree	Disagree	Strongly Disagree

2. There are features I wish the system should possess that are not currently there.

Strongly Agree	Agree	Neither Agree nor Disagree	Disagree	Strongly Disagree

3. I enjoy smooth operation any time I activate the Electronic Health Records Clerking System for daily use.

Strongly Agree	Agree	Neither Agree nor Disagree	Disagree	Strongly Disagree

4. The system can sometimes be slow and hangs and can frustrate your operation for a long time.

Strongly Agree	Agree	Neither Agree nor Disagree	Disagree	Strongly Disagree

5. The system accommodates all patients information needed to comprehensively address their healthcare challenges and I never had need to want to do paper documentation.

Strongly Agree	Agree	Neither Agree nor Disagree	Disagree	Strongly Disagree

6. I can view X-Ray and Ultra Sound images or film in this system.

Strongly Agree	Agree	Neither Agree nor Disagree	Disagree	Strongly Disagree

7. The system has provision or interface that can help the doctor or nurse send requests electronically to other service units like radiology, pharmacy, Laboratory etc, in the hospital.

Strongly Agree	Agree	Neither Agree nor Disagree	Disagree	Strongly Disagree

8. It took me a lot of time to learn how to use this clerking/documentation system.

Strongly Agree	Agree	Neither Agree nor Disagree	Disagree	Strongly Disagree

9. The use of this system really negatively affect the time I need to fully interact with the patient.

Strongly Agree	Agree	Neither Agree nor Disagree	Disagree	Strongly Disagree

10. The use of this electronic clerking/documentation system has increased my daily workload and stress.

Strongly Agree	Agree	Neither Agree nor Disagree	Disagree	Strongly Disagree

11. The use of this new system has negatively affected the number of patients I adequately see in a day.

Strongly Agree	Agree	Neither Agree nor Disagree	Disagree	Strongly Disagree

12. The system is not easy to use that sometimes I am tempted to look for shortcuts around my clerking/documentation challenges.

Strongly Agree	Agree	Neither Agree nor Disagree	Disagree	Strongly Disagree

13. Typing with keyboard all patients demographic, investigations and diagnostic information are all important for a comprehensive care, but I find it difficult typing all with keyboard.

Strongly Agree	Agree	Neither Agree nor Disagree	Disagree	Strongly Disagree

14. I easily know where to find any information I am looking for in this new system.

Strongly Agree	Agree	Neither Agree nor Disagree	Disagree	Strongly Disagree

15. I feel this new system fully satisfied all management and clinicians expectations for introducing it.

Strongly Agree	Agree	Neither Agree nor Disagree	Disagree	Strongly Disagree

16. I enjoy maximum user experience each time I use this system

Strongly Agree	Agree	Neither Agree nor Disagree	Disagree	Strongly Disagree

17. On a scale of 1 to 5 could you choose your best score for this clerking system: **1/5** or **2/5** or **3/5** or **4/5** or **5/5**

where 5/5= **Excellent**	4/5=**Very Good**	3/5=**Good**	2/5=**Poor**	1/5= **Very Poor**

18. Access to internet if generally a huge challenge using this electronic health record clerking system

Strongly Agree	Agree	Neither Agree nor Disagree	Disagree	Strongly Disagree

19. How I wish there could be a faster way of inputting patients health records into this system other than using the keyboard

Strongly Agree	Agree	Neither Agree nor Disagree	Disagree	Strongly Disagree

20. I am almost tempted to think that we are not yet prepared for this new age of electronic health record clerking and documentation.

Strongly Agree	Agree	Neither Agree nor Disagree	Disagree	Strongly Disagree
