# Supplementary material for: The consultant’s guide: evidence-based digital transformation framework for healthcare institutions in developing and resource-constrained economies
Source: BMC Med Inform Decis Mak. 2026 Jul 16;26:405. doi: 10.1186/s12911-026-03713-9 (PMC13659990; doi:10.1186/s12911-026-03713-9)
Supplement: Supplementary file 3 [file 12911_2026_3713_MOESM3_ESM.pdf]

APPENDIX: Manual/Paper Patient Data Capture Forms Currently in Use at the Tertiary/University Teaching Hospitals under Study

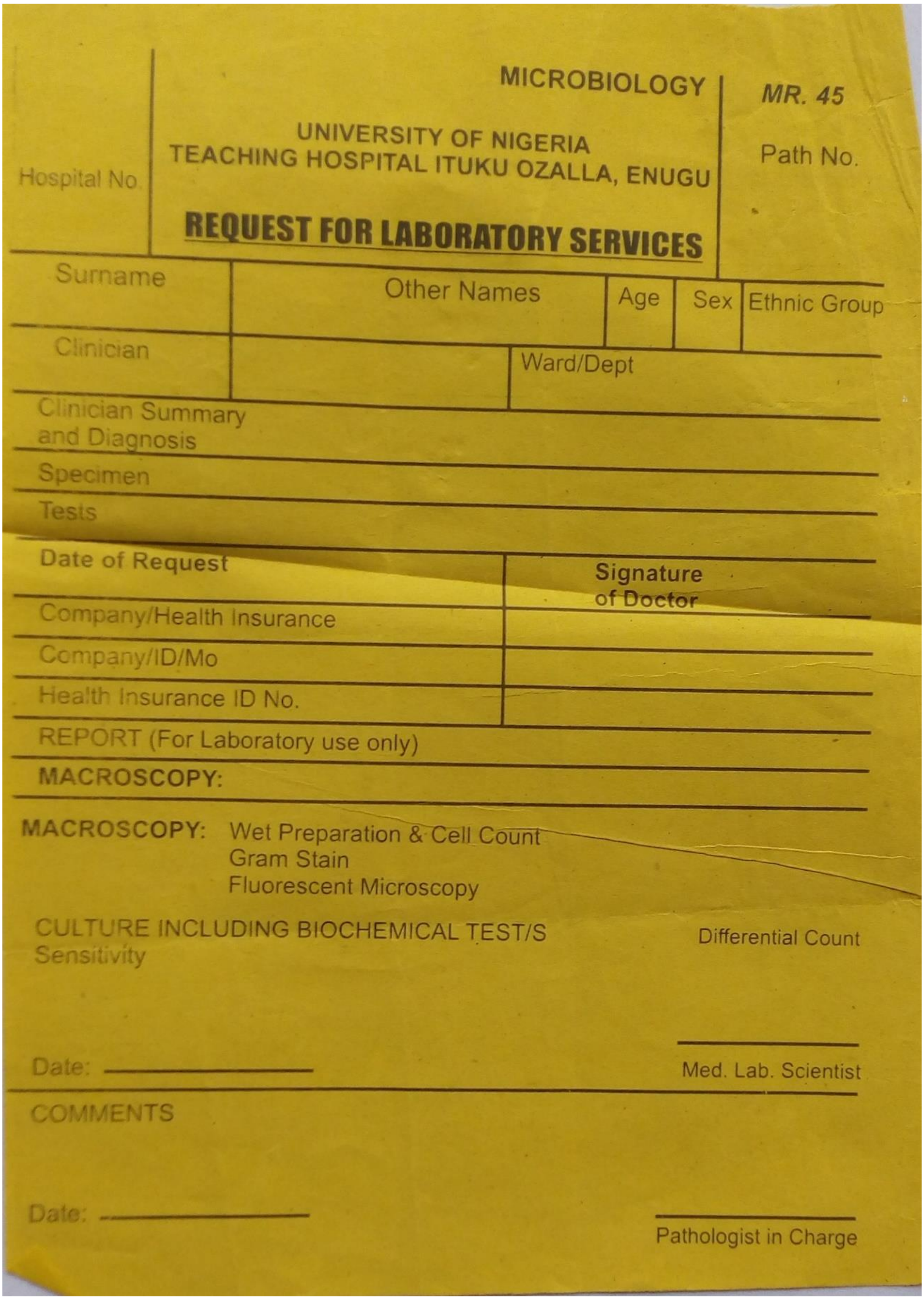

MICROBIOLOGY

MR. 45

Hospital No.

UNIVERSITY OF NIGERIA
TEACHING HOSPITAL ITUKU OZALLA, ENUGU

Path No.

REQUEST FOR LABORATORY SERVICES

Surname	Other Names	Age	Sex	Ethnic Group
Clinician		Ward/Dept		

Clinician Summary and Diagnosis

Specimen

Tests

Date of Request	Signature of Doctor
Company/Health Insurance	
Company/ID/Mo	
Health Insurance ID No.	

REPORT (For Laboratory use only)

MACROSCOPY:

MACROSCOPY: Wet Preparation & Cell Count
Gram Stain
Fluorescent Microscopy

CULTURE INCLUDING BIOCHEMICAL TEST/S
Sensitivity

Differential Count

Date: ____________

Med. Lab. Scientist

COMMENTS

Date: ____________

Pathologist in Charge

Appendix 3 Figure 1: Request Form for Laboratory Services at the University of Nigeria Teaching Hospital

DEPARTMENT OF CHEMICAL PATHOLOGY
UNIVERSITY OF NIGERIA TEACHING HOSPITAL, ITUKU OZALLA, ENUGU

LABORATORY REQUEST AND REPORT FORM

MR. 43

Patient's Name (Block Letters)<br>Surname<br>First Name (s)	Unit No<br>GP.<br>Cas/Ward/Clinic	Clinical Details and Present Medication

Address/Postal Address | Tel: | E-mail:

Age | Date of Birth D M Y | Sex | Tribe | Nationality | Religion

Nature of Specimen | Fasting ( ) ( ) Yes ( ) No | Time Collected | Date Collected | L.M.P.

Examination Required | Referring Consultant & Sign

Company/Wealth Insurance

Company I D #

Health Insurance I D #

Receipt No. | LABORATORY REPORT (for lab use only) Lab. Ref. No.

TESTS AND UNITS

CHEMISTRY			
RENAL/ELECTORYTES/BONE		CON. UNIT	RESULT
SODIUM	mmoL/L	135-145	
POTASSIUM	mmoL/L	3.5-5.0	
BICARBONATE	mmoL/L	24.28	
CHLORIDE	mmoL/L	97.108	
UREA	mmoL/L	2.5-8.5	
CREATINE	mmoL/L	44.2-194.5	
URIC ACID	mmoL/L	0.08-c.4	
URIC ACID (24 HR URINE)	mmoL/L		
CALCIUM	mmoL/L	2.2-2.8	
MAGNESIUM	mmoL/L	0.72	
INORGANIC PHOSPHORUS	mmoL/L	1.1-1.8	
CALCIUM/PHOSPHATE (24 HR. URINE)	mmoL/L		
PROTEIN (24 HR. URINE)	gL		
CALCULUS ANALYSIS			
LIVER/PANCREAS			
BILIRUBIN (TOTAL)	umo L/L	8.0-17.0	
BILIRUBIN (DIRECT)	umo L/L	Less than 8.0	
AIP	U/L	25 - 92	
AST	U/L	5 - 15	
ALT	U/L	3 - 15	
PROTEIN (TOTAL)	g/L	60 - 80	
ALBUMIN	g/L	30 - 50	
GLOBULIN	g/L	18 - 30	
CAERULOPIASMIN	mg/L	300 - 600	
SERUM PROTEIN ELECTROPHERESIS (SPE)			
AMYLASE (SERUM)	U/L	130 - 320	
AMYLASE (URINE)	U/L		
LIPASE	U/L		
GGT (MALE)	U/L	<50	
GGT (FEMALE)	U/L	<30	
DIABETES			
GLUCOSE (FASTING)	mmoL/L	3.6 - 5.7	
CLUCOSE (RANDON)	mmoL/L	3.6 - 6.7	
GLUCOSE (2HR. PP)	mmoL/L		
GIT (21/2 HRS)	mmoL/L		
HBAIC	%	5 - 8	
MICROAL BUMIN (URINE)	mmoL/L		
CARDIAC MARKERS			
CK (MALE)	U/L	5 - 55	
CK (FEMALE)		5 - 36	
CK MB	U/L	Less than 6	
MYOGLOBIN (MALE)	ng/mL	16 - 76	
MYOGLOBIN (FEMALE)	ng/mL	7 - 64	
C. TROPONIN .T	ng/mL	Less than 0.03	
TUMOUR MARKERS			
PSA	ng/mL	Less than 0.40	
CEA (GIT, LUNG BREAST			
CO. 15-3 (BREAST)			
CA 19-9 (G.I.T. PANCREAS)			
AFP	U/L	<20	
B-2 MICROGLOBUUN			
BENCE. JONES PROTEIN			
ACID PHOSPHATASE (TOTAL)	U/L	0.9 - 9.1	
ACID PHOSPHATASE (PROSTATIC)	U/L	<1.0	

ENDCORINOLOGY		CON. UNIT	RESULT
A. THROID			
TSH	U/L	0.2 - 5.5	
T4 - Total	mmoL/L	55 - 140	
T4 (Free T4)	PmoL/L	9 - 23	
T3 - Total	mmoL/L	1.3 - 3.4	
T3 (Free T3)	PmoL/L	3.0 - 8.6	
B. REPRODUCTIVE ENDOCRINOLOGY			
FSH	mU/L	2.18	
FSH (mid cycle)	mU/L	12.30	
LH (Basal)	mU/L	6 - 60	
LH (mid cycle)	mU/L	60 - 220	
17/B-Estradiol (fulllcular)		0.1 - 0.26	
17/B-Estradiol (mid cycle)	nmol/L	0.35-1.50	
17/B-Estradiol(Luleal)	nmol/L	0.2 - 1.10	
17/B-Estradiol Postmenopausal		<0.2	
Progesterone	nmol/L	<44	
17 - OH Progesterone	nmol/L	<15	
Testosterone (male)	nmol/L	10 - 30	
Testosterone (female)	nmol/L	0.3 - 25	
Prolactin	ng/L	<20	
C. ADRENAL/ & OTHERS			
Cortisol	nmol/L	280-700	
Cortisol (24 Hr Urine)	nmol/L	150-900	
Growth Hormone (Basa)	mU/L	<10	
Insulin	mU/L	<10	
VMA (24 hr urine)	umol/24hr	20	
5 - HIAA (24 hr urine)	umol/24hr	30	
CSF Protein	g/L	0.15-04	
CSF Glucose	mmol/L	2.5-5.0	
Genetic Screens			
DNA Poternity Testing			
LIPIDS/CHD RISK			
CHOLESTEROL (Total)	mmol/L	3.6-6.2	
HDL - C	mmol/L	0.8-17	
LDL - C	mmol/L	1.9-3.6	
VLDL	mmol/L	0.4-1.3	
TRIGLYCERIDE	mmol/L	0.5-1.0	
C R P	mg/ML		
HOMOCYSTEINE			
D - DIMER	mg/ML	<0.5	
LP ELECTROPHORESIS			
PREGNANCY TEST			
G 6 PD			
IRON	mmol/L	13 - 32	
TIBC	mmol/L	45 - 70	
IgM	g/L	0.6-2.5	
IgA	g/L	0.9-4.5	
IgE	g/L		
IgG	g/L	7 - 18	
On Special request			

Appendix 3 Figure 2: Laboratory Request and Report Form at the University of Nigeria Teaching Hospital

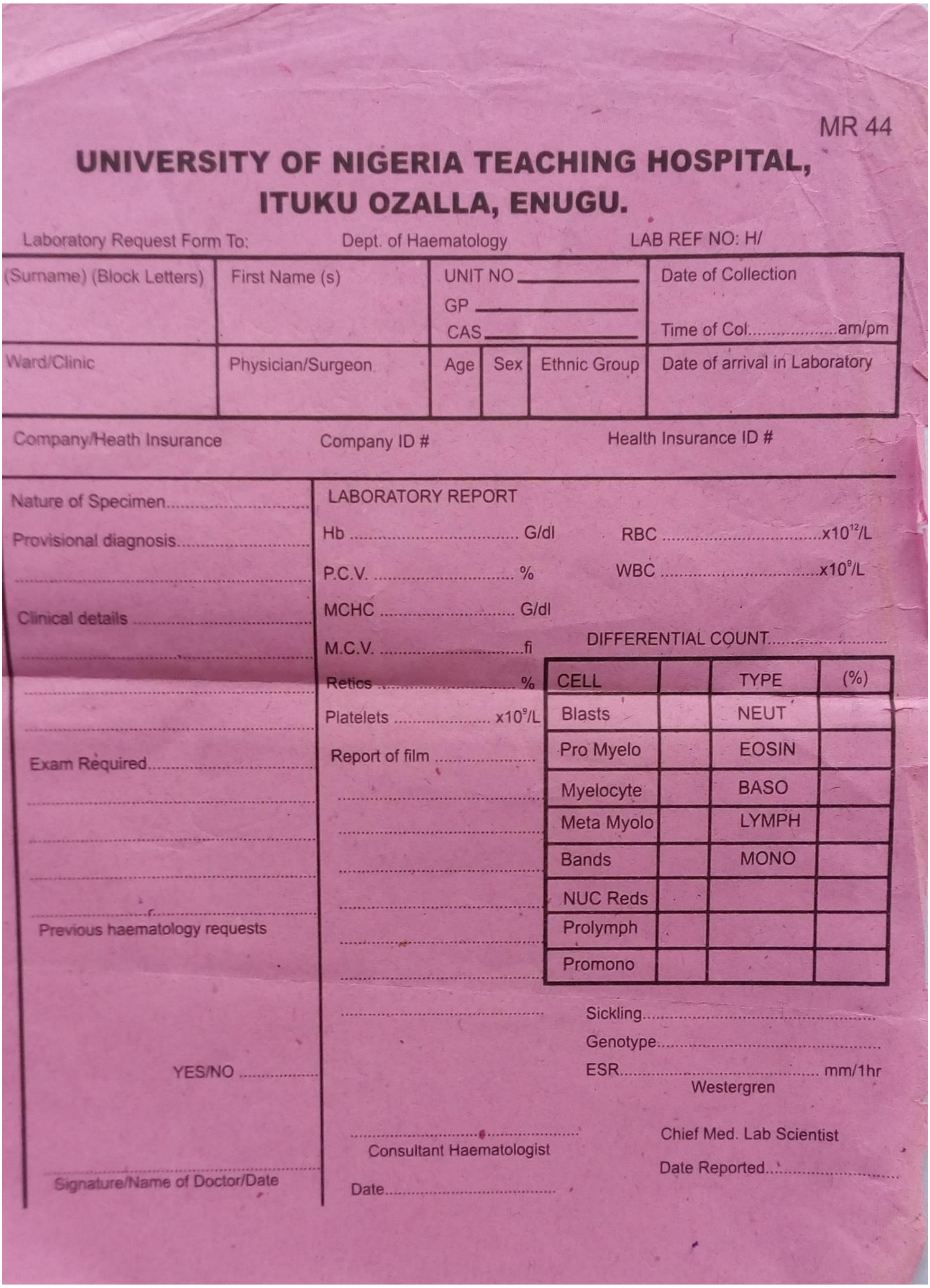

MR 44

UNIVERSITY OF NIGERIA TEACHING HOSPITAL,
ITUKU OZALLA, ENUGU.

Laboratory Request Form To: Dept. of Haematology LAB REF NO: H/

(Surname) (Block Letters)	First Name (s)	UNIT NO ______<br>GP ______<br>CAS ______			Date of Collection<br>Time of Col.................am/pm
Ward/Clinic	Physician/Surgeon	Age	Sex	Ethnic Group	Date of arrival in Laboratory

Company/Heath Insurance Company ID # Health Insurance ID #

Nature of Specimen..............................

Provisional diagnosis...........................

....

Clinical details ....

....

....

....

Exam Required...................................

....

....

....

....

Previous haematology requests

YES/NO ....

....
Signature/Name of Doctor/Date

LABORATORY REPORT

Hb .... G/dl RBC ....x10^{12}/L

P.C.V. .... % WBC ....x10^{9}/L

MCHC .... G/dl

M.C.V. ....fi DIFFERENTIAL COUNT.......................

Retics .... %

Platelets .... x10^{9}/L

Report of film ....

CELL		TYPE	(%)
Blasts		NEUT	
Pro Myelo		EOSIN	
Myelocyte		BASO	
Meta Myolo		LYMPH	
Bands		MONO	
NUC Reds			
Prolymph			
Promono			

Sickling...

Genotype..

ESR.. mm/1hr
Westergren

....
Consultant Haematologist

Date.....................................

Chief Med. Lab Scientist

Date Reported..........................

Appendix 3 Figure 3: Laboratory Report Form for University of Nigeria Teaching Hospital

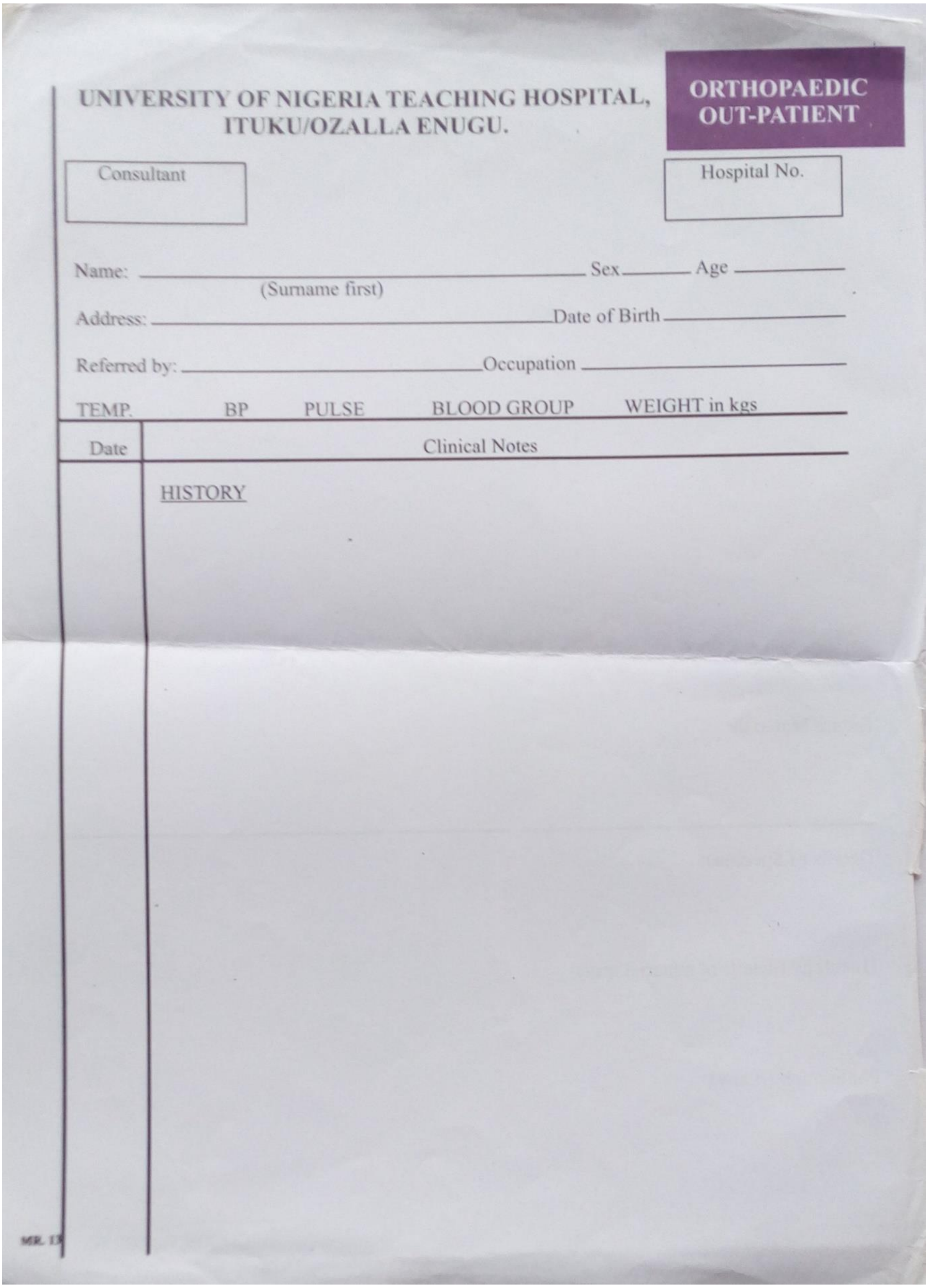

UNIVERSITY OF NIGERIA TEACHING HOSPITAL,
ITUKU/OZALLA ENUGU.

ORTHOPAEDIC
OUT-PATIENT

Consultant

Hospital No.

Name: ______________________ Sex ______ Age ______
(Surname first)

Address: ______________________ Date of Birth ______

Referred by: ______________________ Occupation ______

TEMP. BP PULSE BLOOD GROUP WEIGHT in kgs

Date	Clinical Notes
	HISTORY

Appendix 3 Figure 4: Clinical Notes Form at the University of Nigeria Teaching Hospital

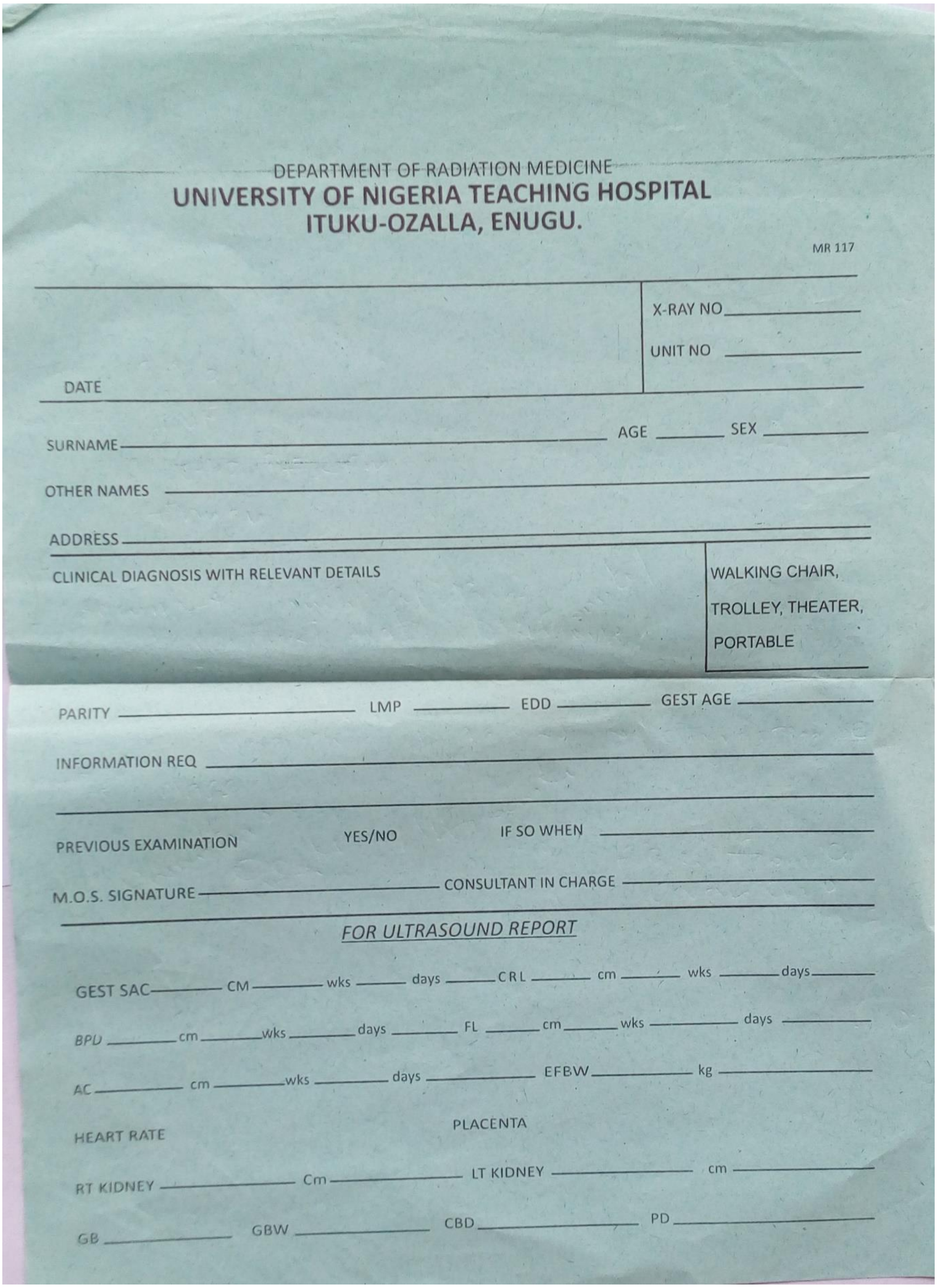

DEPARTMENT OF RADIATION MEDICINE
UNIVERSITY OF NIGERIA TEACHING HOSPITAL
ITUKU-OZALLA, ENUGU.

MR 117

X-RAY NO
UNIT NO
DATE

SURNAME AGE SEX
OTHER NAMES
ADDRESS

CLINICAL DIAGNOSIS WITH RELEVANT DETAILS

WALKING CHAIR, TROLLEY, THEATER, PORTABLE

PARITY LMP EDD GEST AGE
INFORMATION REQ
PREVIOUS EXAMINATION YES/NO IF SO WHEN
M.O.S. SIGNATURE CONSULTANT IN CHARGE

FOR ULTRASOUND REPORT

GEST SAC CM wks days C R L cm wks days
BPD cm wks days FL cm wks days
AC cm wks days EFBW kg
HEART RATE PLACENTA
RT KIDNEY Cm LT KIDNEY cm
GB GBW CBD PD

Appendix 3 Figure 5: Ultrasound Report Form at the University of Nigeria Teaching Hospital

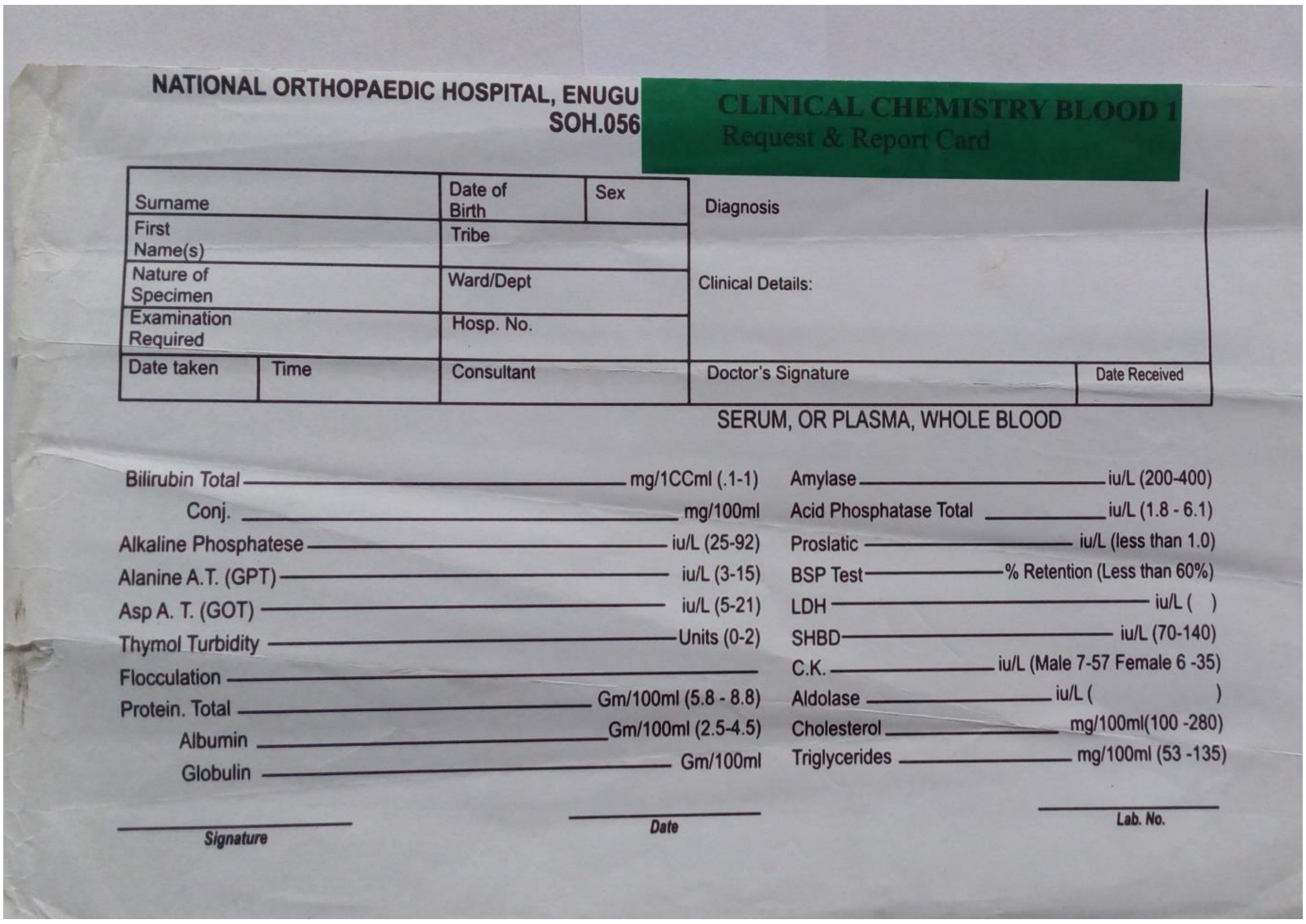

NATIONAL ORTHOPAEDIC HOSPITAL, ENUGU
SOH.056

CLINICAL CHEMISTRY BLOOD 1
Request & Report Card

Surname	Date of Birth	Sex	Diagnosis
First Name(s)	Tribe		
Nature of Specimen	Ward/Dept		Clinical Details:
Examination Required	Hosp. No.		
Date taken / Time	Consultant		Doctor's Signature / Date Received

SERUM, OR PLASMA, WHOLE BLOOD

Bilirubin Total ________ mg/1CCml (.1-1)
Conj. ________ mg/100ml
Alkaline Phosphatese ________ iu/L (25-92)
Alanine A.T. (GPT) ________ iu/L (3-15)
Asp A. T. (GOT) ________ iu/L (5-21)
Thymol Turbidity ________ Units (0-2)
Flocculation ________
Protein. Total ________ Gm/100ml (5.8 - 8.8)
Albumin ________ Gm/100ml (2.5-4.5)
Globulin ________ Gm/100ml

Amylase ________ iu/L (200-400)
Acid Phosphatase Total ________ iu/L (1.8 - 6.1)
Proslatic ________ iu/L (less than 1.0)
BSP Test ________ % Retention (Less than 60%)
LDH ________ iu/L ( )
SHBD ________ iu/L (70-140)
C.K. ________ iu/L (Male 7-57 Female 6 -35)
Aldolase ________ iu/L ( )
Cholesterol ________ mg/100ml(100 -280)
Triglycerides ________ mg/100ml (53 -135)

Signature ________ Date ________ Lab. No. ________

Appendix 3 Figure 6: Clinical Chemistry Blood Report Form at the National Orthopaedic Hospital Enugu

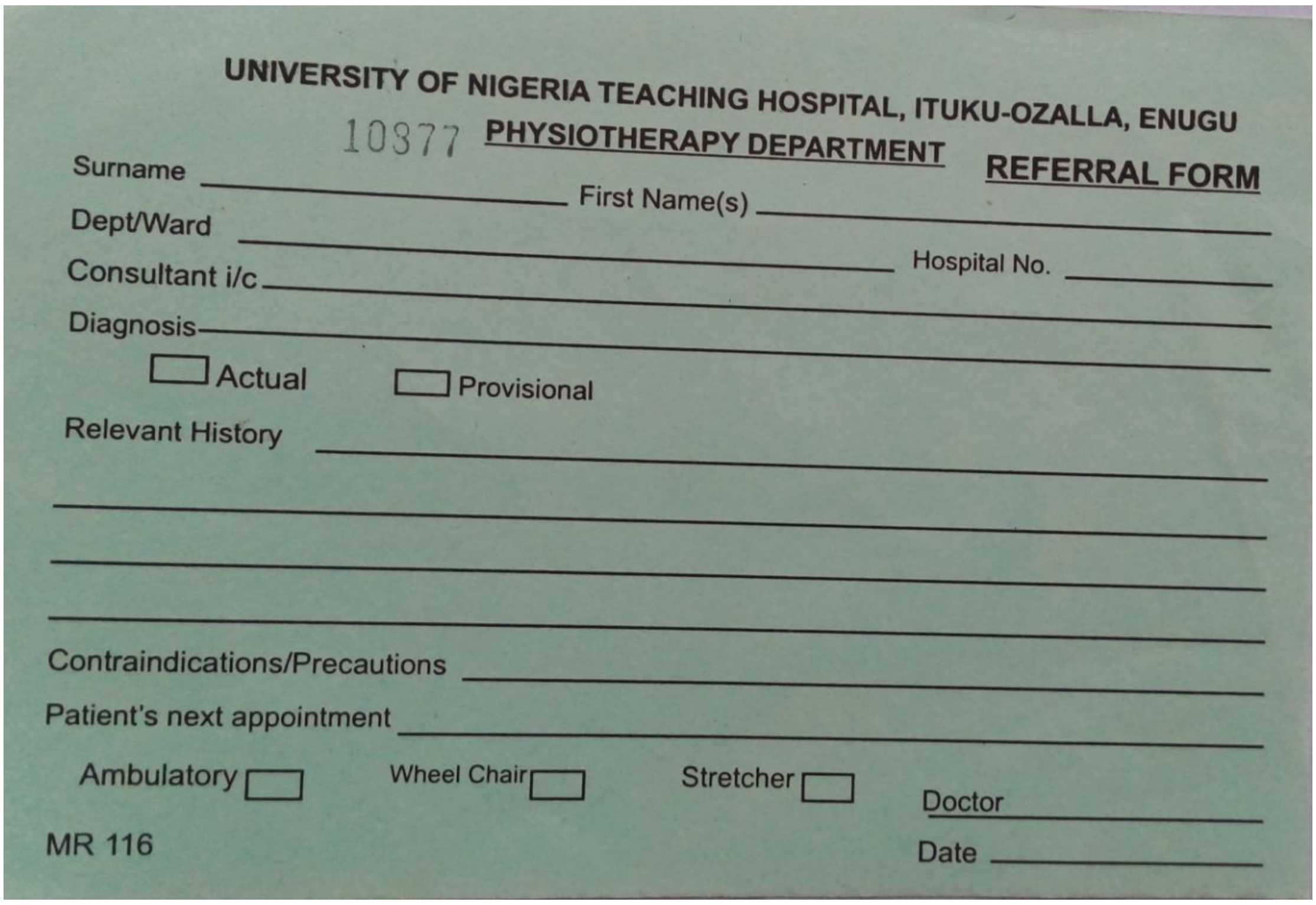

UNIVERSITY OF NIGERIA TEACHING HOSPITAL, ITUKU-OZALLA, ENUGU
10377 PHYSIOTHERAPY DEPARTMENT
REFERRAL FORM

Surname ________ First Name(s) ________
Dept/Ward ________ Hospital No. ________
Consultant i/c ________
Diagnosis ________
☐ Actual ☐ Provisional
Relevant History ________
Contraindications/Precautions ________
Patient's next appointment ________
Ambulatory ☐ Wheel Chair ☐ Stretcher ☐
Doctor ________
MR 116
Date ________

Appendix 3 Figure 7: Physiotherapy Referral Form at the University of Nigeria Teaching Hospital

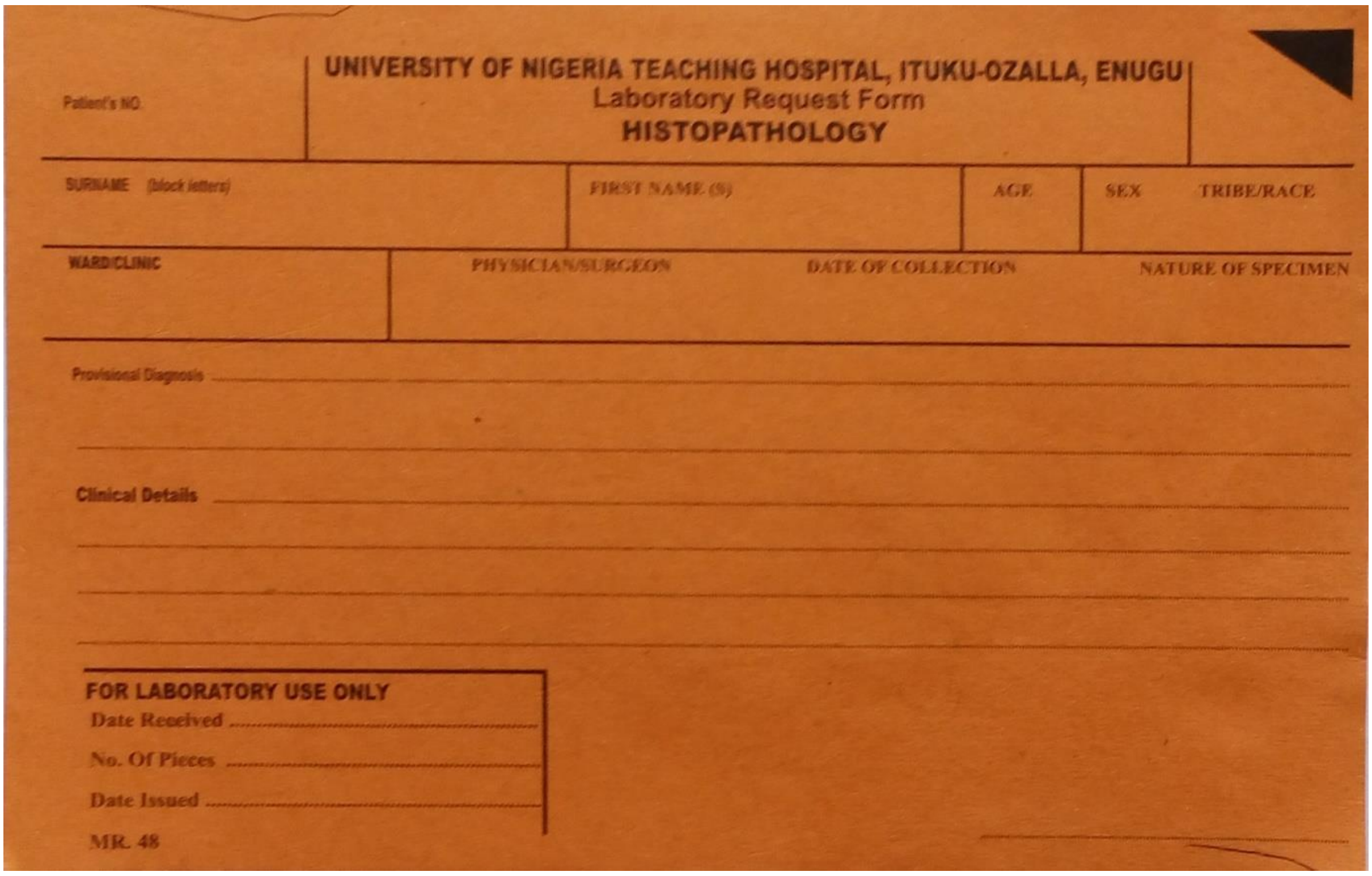

UNIVERSITY OF NIGERIA TEACHING HOSPITAL, ITUKU-OZALLA, ENUGU
Laboratory Request Form
HISTOPATHOLOGY

Patient's NO.

SURNAME (block letters) | FIRST NAME (S) | AGE | SEX | TRIBE/RACE

WARD/CLINIC | PHYSICIAN/SURGEON | DATE OF COLLECTION | NATURE OF SPECIMEN

Provisional Diagnosis

Clinical Details

FOR LABORATORY USE ONLY
Date Received
No. Of Pieces
Date Issued
MR. 48

Appendix 3 Figure 8: Histopathology Laboratory Request Form at the University of Nigeria Teaching Hospital

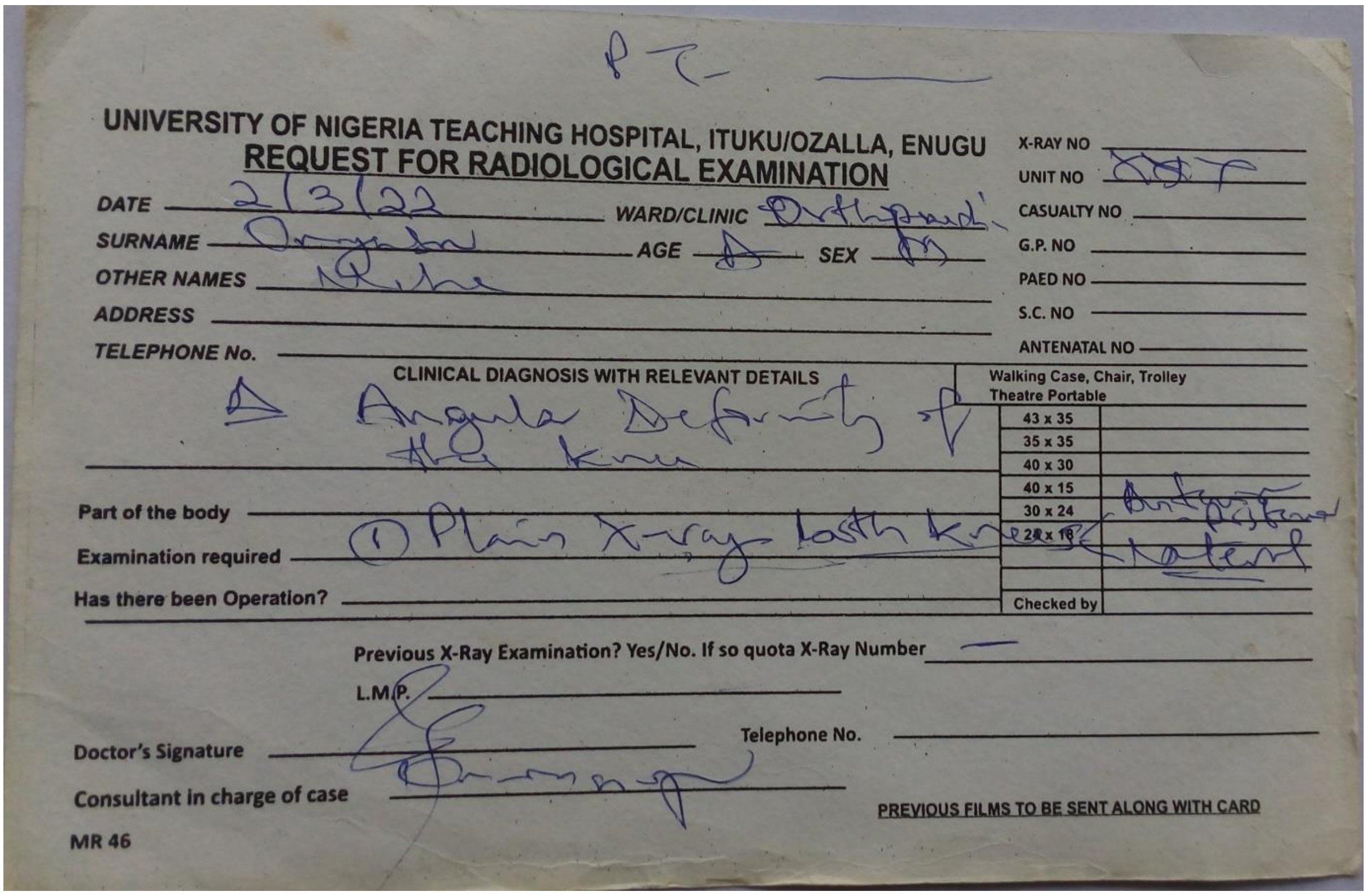

UNIVERSITY OF NIGERIA TEACHING HOSPITAL, ITUKU/OZALLA, ENUGU
REQUEST FOR RADIOLOGICAL EXAMINATION

DATE 2/3/22
WARD/CLINIC Orthopaedic
SURNAME
AGE
SEX
OTHER NAMES
ADDRESS
TELEPHONE No.

X-RAY NO
UNIT NO
CASUALTY NO
G.P. NO
PAED NO
S.C. NO
ANTENATAL NO

CLINICAL DIAGNOSIS WITH RELEVANT DETAILS
Angular Deformity of the knee

Walking Case, Chair, Trolley
Theatre Portable
43 x 35
35 x 35
40 x 30
40 x 15
30 x 24
24 x 18
Checked by

Part of the body
Examination required (1) Plain X-ray both knee — Antero-posterior, lateral
Has there been Operation?

Previous X-Ray Examination? Yes/No. If so quota X-Ray Number
L.M.P.
Doctor's Signature
Telephone No.
Consultant in charge of case
PREVIOUS FILMS TO BE SENT ALONG WITH CARD
MR 46

Appendix 3 Figure 9: Request Form for Radiological Examination at the University of Nigeria Teaching Hospital
